# Reassessment of the possible size, form, weight, cruising speed, and growth parameters of the extinct megatooth shark, *Otodus megalodon* (Lamniformes: Otodontidae), and new evolutionary insights into its gigantism, life history strategies, ecology, and extinction

**DOI:** 10.26879/1502

**Published:** 2025

**Authors:** Kenshu Shimada, Ryosuke Motani, Jake J. Wood, Phillip C. Sternes, Taketeru Tomita, Mohamad Bazzi, Alberto Collareta, Joel H. Gayford, Julia Türtscher, Patrick L. Jambura, Jürgen Kriwet, Romain Vullo, Douglas J. Long, Adam P. Summers, John G. Maisey, Charlie Underwood, David J. Ward, Harry M. Maisch, Victor J. Perez, Iris Feichtinger, Gavin J.P. Naylor, Joshua K. Moyer, Timothy E. Higham, João Paulo C.B. da Silva, Hugo Bornatowski, Gerardo González-Barba, Michael L. Griffiths, Martin A. Becker, Mikael Siversson

**Affiliations:** Department of Biological Sciences, DePaul University, Chicago, Illinois, USA, Department of Environmental Science and Studies DePaul University, Chicago, Illinois, USA, and Sternberg Museum of Natural History, Fort Hays State University, Hays, Kansas, USA; Department of Earth and Planetary Sciences, University of California Davis, Davis, California, USA; Department of Biological Sciences, Florida Atlantic University, Boca Raton, Florida, USA; Education and Conservation Department, SeaWorld, San Diego, CA, USA, and Shark Measurements, London, UK; Okinawa Churashima Research Center, Okinawa Churashima Foundation, Motobu-cho, Okinawa, Japan and Okinawa Churaumi Aquarium, Okinawa Churashima Foundation, Motobu-cho, Okinawa, Japan; Department of Earth and Planetary Sciences, Stanford University, Stanford, California, USA; Dipartimento di Scienze della Terra, Università di Pisa, Pisa, PI, Italy; Department of Marine Biology and Aquaculture, James Cook University, Townsville, Australia, and Shark Measurements, London, UK; Department of Palaeontology, University of Vienna, Vienna, Austria; Department of Palaeontology, University of Vienna, Vienna, Austria; Department of Palaeontology, University of Vienna, Vienna, Austria and Vienna Doctoral School of Ecology and Evolution (VDSEE), University of Vienna, Vienna, Austria; Université de Rennes, CNRS, Géosciences Rennes, UMR 6118, Rennes, France; Department of Ichthyology, California Academy of Sciences, San Francisco, California, USA; Friday Harbor Laboratories, Department of Biology and SAFS, University of Washington, Seattle, Washington, USA; Department of Vertebrate Paleontology, American Natural History Museum, New York, New York, USA; School of Natural Sciences, Birkbeck College, London, UK; Department of Earth Sciences, Natural History Museum, London, UK; Department of Marine and Earth Sciences, Florida Gulf Coast University, Fort Myers, Florida, USA; Environmental Studies Department, St. Mary’s College of Maryland, St. Mary’s City, Maryland, USA; Geological-Palaeontological Department, Natural History Museum, Vienna, Austria; Florida Museum of Natural History, University of Florida, Gainesville, Florida, USA; Department of Ecology and Evolutionary Biology, Yale University, New Haven, Connecticut, USA, and Atlantic Shark Institute, Wakefield, Rhode Island, USA; Department of Evolution, Ecology, and Organismal Biology, University of California Riverside, Riverside, California, USA; Departamento de Sistemática e Ecologia, Centro de Ciências Exatas e da Natureza, Universidade Federal da Paraíba, Castelo Branco, João Pessoa, PB, Brazil; Center for Marine Studies, Universidade Federal do Paraná, Brazil; Museo de Historia Natural- UABCS, Colonia El Mezquitito, CP, La Paz, Baja California Sur, Mexico; Department of Environmental Science, William Paterson University of New Jersey, Wayne, New Jersey, USA; Department of Environmental Science, William Paterson University of New Jersey, Wayne, New Jersey, USA; Department of Earth and Planetary Sciences, Western Australian Museum, Welshpool, WA, Australia, and School of Molecular and Life Sciences, Curtin University, Bentley, WA, Australia

**Keywords:** body length, body mass, body reconstruction, Neogene, ontogeny

## Abstract

*Otodus megalodon* (Lamniformes: Otodontidae) is an iconic Neogene shark, but the lack of well-preserved skeletons has hampered our understanding of various aspects of its biology. Here, we reassess some of its biological properties using a new approach, based on known vertebral specimens of *O. megalodon* and 165 species of extinct and extant neoselachian sharks across ten orders. Using the median neurocranial and caudal fin proportions relative to the trunk proportion among non-mitsukurinid/non-alopiid lamniforms, we show that *O. megalodon* could have had a slender body and possibly reached about 24.3 m in length. Allometric considerations indicate that a stout body plan like the extant white shark *(Carcharodon carcharias)* for *O. megalodon* could have incurred excessive hydrodynamic costs, further supporting the interpretation that *O. megalodon* likely had a slenderer body than *C. carcharias*. A 24.3-m-long *O. megalodon* may have weighed around 94 t, with an estimated cruising speed of 2.1–3.5 km h^-1^. A reanalysis of vertebral growth bands suggests a size at birth of 3.6–3.9 m for *O. megalodon*, supporting the previous interpretations of its ovoviviparity and embryos’ intrauterine oophagous behavior, but less likely the need for nursery areas. Additional inferred growth patterns corroborated by the known fossil record support the hypothesis that the emergence of *C. carcharias* during the Early Pliocene is at least partly responsible for the demise of *O. megalodon* due to competition for resources. These interpretations are working hypotheses expected to serve as reasonable reference points for future studies on the biology of *O. megalodon*.

## Introduction

**Figure 1:**
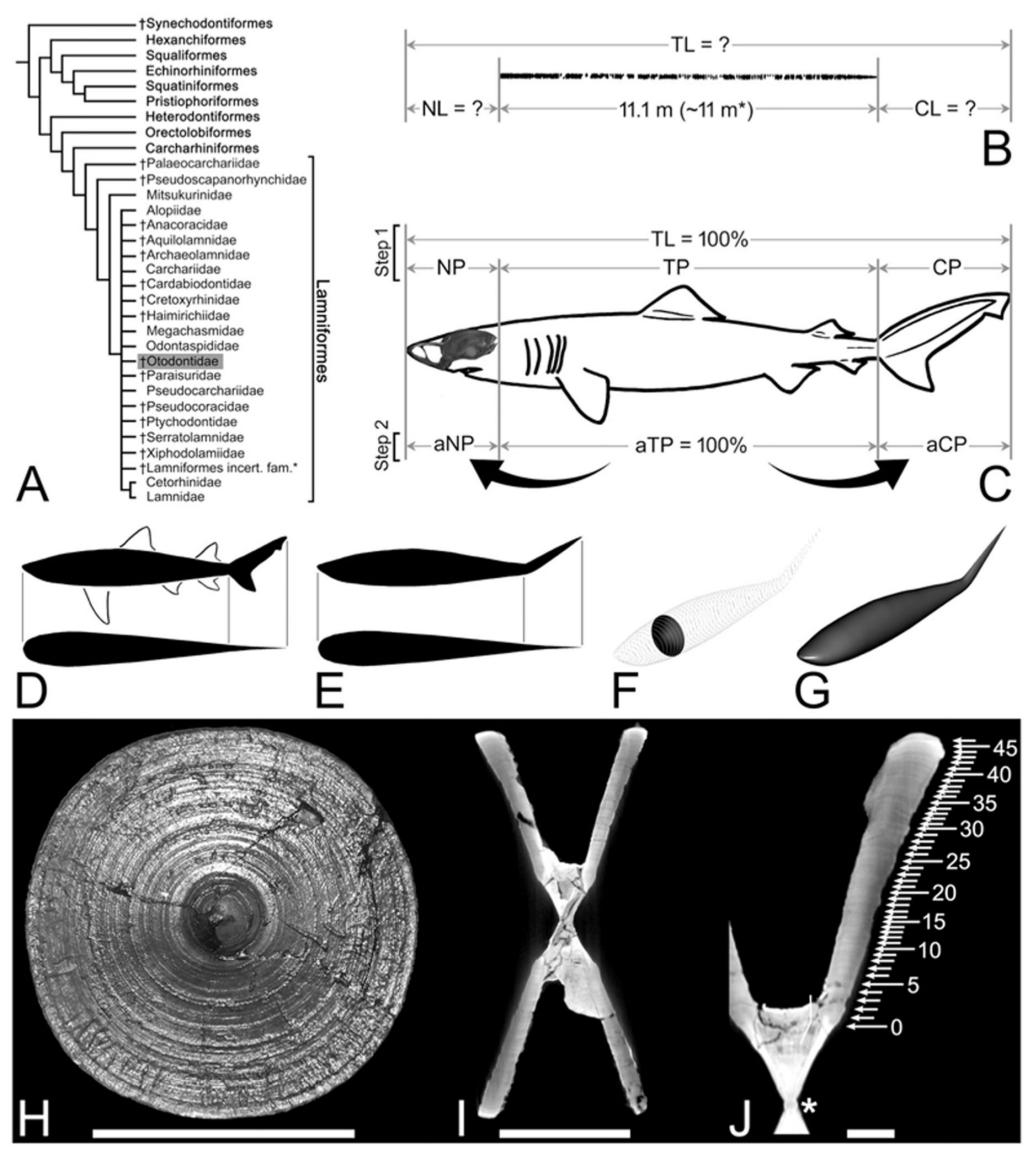
Background information. **A**, Conceptualized current understanding of family-level phylogeny of Lamniformes along with nine other neoselachian shark orders discussed in this study (ten orders are in bold; the family †Otodontidae that includes †*Otodus megalodon* is highlighted in gray box; category with an asterisk [*] includes several genera of uncertain familial placements, such as †*Priscusurus* and †*Trigonotodus*: see Cappetta, 2012; sources of information: Klug, 2010; Cappetta, 2012; Vullo et al., 2016, 2021, 2024; Landemaine et al., 2018; Shimada and Everhart, 2019; Jambura et al., 2019, 2023; Stone and Shimada, 2019; Sternes et al., 2023, 2024; Silva et al., 2023; note that, although neoselachians, the monophyly and composition of †Synechodontiformes are questionable: see Maisey, 2012; the lamniform attribution of †*Palaeocarcharias stromeri* is tentative: see Villalobos-Segura et al., 2023; Guinot et al., 2025). **B**, Silhouette of reconstructed vertebral column of †*Otodus megalodon* based on associated vertebral set from the Miocene of Belgium (IRSNB P 9893) and its total measured length by Cooper et al. (2022), where the total length (TL) of the shark is unknown because its neurocranial length (NL) and caudal fin length (CL) are unknown (asterisk [*] indicates the vertebral column length used in this study: see text). **C**, Schematic illustration using extant *Pseudocarcharias kamoharai* as an example showing that Step 1 of this study investigates the neurocranial proportion (NP), trunk proportion (TP), and caudal fin proportion (CP) in relation to TL, whereas Step 2 shows that the “adjusted neurocranial proportion” (aNP) and “adjusted caudal fin proportion” (aCP) are determined by considering TP as 100% (or “adjusted trunk proportion” [aTP]) (line drawing based on Ebert et al., 2021, p. 307; neurocranial image based on CT scan rendering of FMNH 117474). **D**, Example of silhouettes of a shark in lateral (top) and dorsoventral (bottom) views for body weight (body mass) estimation (see E–G; this example depicts *Negaprion brevirostris* [lemon shark] based largely on Ebert et al., 2021, p. 561: see text). **E**, Example silhouette in lateral (top) and dorsoventral (bottom) views with non-caudal fins as well as non-muscular portions of the caudal fin removed (after D) for body weight (body mass) estimation (see F). **F**, Serial superelliptical sections generated based on example silhouettes in E. **G**, 3D mesh combining all superelliptical slices as in F. **H**, One of the largest vertebrae (“centrum #1”) of †*Otodus megalodon* in IRSNB P 9893 (see B; scale bar equals 10 cm; photograph courtesy of IRSNB). **I**, Computed tomographic image showing a sagittal cross-sectional view of vertebra depicted in H (scale bar equals 5 cm; after Shimada et al., 2021b, figure 1b). **J**, Computed tomographic image of a sagittal cross-sectional view of the largest vertebrae (“centrum #4”) in IRSNB P 9893 showing incremental growth bands presumably formed annually based on Shimada et al. (2021b, figure 1b) (scale bar equals 1 cm; * = center of vertebra; bent line = “angle of change”: see Shimada et al., 2021b).

Body size impacts various aspects of the life and mortality risks of every animal, including the outcome of ecological interactions with other organisms (e.g., predation vulnerability and foraging success), dispersal capabilities and speed, energy reserve storage capacity, body heat retention capacity, and tolerance of environmental change (e.g., Bergmann, 1847; Peters, 1986; Kram and Taylor, 1990; Cohen et al., 1993; Hone and Benton, 2005; Speakman, 2005; Brown and Sibly, 2006; Healy et al., 2013). Assessments of the body size of large extinct carnivores typically include elucidation of their life history traits such as growth parameters and patterns, and the ecological niches they may have filled (Cailliet and Goldman, 2004; Goldman et al., 2012; Shimada et al., 2021b). Yet, deciphering these key biological properties, sometimes as simple as body size itself, can be difficult, particularly for species that are known from incomplete fossil specimens (e.g., Gayford et al., 2024b, and references therein). The iconic prehistoric shark †*Otodus megalodon* (Lamniformes: †Otodontidae) is an excellent example of such a taxon, the biological properties of which have long been the subject of debate in the scientific literature (Figure 1A; dagger [†] symbol indicates extinct).

†*Otodus megalodon* is represented primarily by its gigantic teeth measuring up to at least 16 cm and possibly as much as about 20 cm in height from Neogene (specifically mid-Miocene–Early Pliocene) marine deposits nearly worldwide (Cappetta, 2012; Pimiento et al., 2016; Shimada, 2019; Pollerspöck et al., 2023). Some vertebrae, placoid scales, and fragments of tessellated cartilage of †*O. megalodon* have also been reported up to now (e.g., Bendix-Almgreen, 1982, 1983; Uyeno and Sakamoto 1984; Gottfried et al., 1996; Kent, 2018; Cooper et al., 2022; Shimada et al., 2024a, 2024b). However, the lack of complete fossil specimens has resulted in uncertainty regarding the true size of this prehistoric shark (Sternes et al., 2023, 2024). This paucity of fossil material has hampered our understanding of the biology and ecology of †*O. megalodon*, despite its presumed significant role in shaping the modern-day marine ecosystem as one of the largest carnivores that ever existed (Shimada, 2019; Shimada et al., 2024a).

Previously, the total length (TL: see Ebert et al., 2021, figure 68) of †*Otodus megalodon* was estimated based on comparisons between the vertebral or tooth sizes of †*O. megalodon* with those of the extant white shark, *Carcharodon carcharias* (Randall, 1973; Gottfried et al., 1996; Shimada, 2003, 2019; Perez et al., 2021). These studies estimated the maximum TL of †*O. megalodon* to be at least 15 m and as much as about 20 m (e.g., Shimada, 2019; Perez et al., 2021), with a TL at birth of about 2 m (Shimada et al., 2021b). Likewise, estimates of body weight (BW) for †*O. megalodon* were conducted using the extant white shark as a proxy, with maximum estimates ranging up to about 103 metric tons (t) for a hypothetical 20.3-m-TL individual (Gottfried et al., 1996; Cooper et al., 2022). The use of *C. carcharias* as a modern analog for †*O. megalodon* was historically considered logical, particularly in earlier studies (e.g., Randall, 1973; Gottfried et al., 1996). This is because the species was assigned to the genus *Carcharodon* (Lamnidae) with the interpretation that *“Carcharodon” megalodon* was the direct ancestor or a sister taxon of the extant *C. carcharias* due to their large, triangular serrated teeth (e.g., Applegate and Espinosa-Arrubarrena, 1996; Purdy, 1996). Whilst †*O. megalodon* is now generally considered to belong to †*Otodus* within the extinct lamniform family †Otodontidae rather than *Carcharodon* or Lamnidae (Cappetta, 2012; Shimada et al., 2017; Figure 1A), the use of extant *C. carcharias* or other extant lamnids as a proxy to infer the body size or other aspects of the biology of †*O. megalodon* has continued simply because of a perceived lack of any suitable modern alternatives (e.g., Reolid and Molina, 2015; Razak and Kocsis, 2018; Shimada, 2019; Perez et al., 2021; Cooper et al., 2020, 2022).

However, the practice of using extant *Carcharodon carcharias* or other lamnids to infer the biology of †*Otodus megalodon*, including its body size and form, has recently been called into question (Sternes et al., 2023, 2024). Sternes et al. (2024) pointed out that the total combined length of an incomplete vertebral column of †*O. megalodon* from the Miocene of Belgium (IRSNB P 9893 housed in the Royal Belgian Institute of Natural Sciences in Brussels) mostly consisting of trunk vertebrae was 11.1 m (Cooper et al., 2022; Figure 1B), but the same fossil individual was previously estimated to be 9.2 m TL (Gottfried et al., 1996). This apparently contradictory estimate included the head and the caudal fin and was based on the comparison of vertebral diameters in 16 individuals of the extant *C. carcharias* (Gottfried et al., 1996). This discrepancy indicates that the extant lamnids, including *C. carcharias*, may not serve as appropriate modern analogs for †*O. megalodon* as they most likely result in underestimated TL values for †*O. megalodon*. In point of fact, Sternes et al. (2024) suggested that †*O. megalodon* likely had a slenderer body than the extant white shark.

The corollary of Sternes et al.’s (2024) study is that it is better not to make any a priori assumption that any single extant shark taxon (e.g., *Carcharodon carcharias* or any other lamnid) would provide adequate estimates of †*Otodus megalodon*’s biological parameters. Therefore, with the assumption that IRSNB P 9893 largely represents the entire length of the trunk vertebrae, here we take a novel approach to reassess the body length of †*O. megalodon*. Specifically, we survey the proportional relationship of the trunk length to the neurocranial length (NL) as well as that of the trunk length to the caudal fin length (CL) (Figure 1B) across a wide range of Mesozoic–Cenozoic neoselachians to infer the anteroposterior length of the neurocranium (a proxy for the head length) and the caudal fin in †*O. megalodon*. Based on the newly estimated TL, we reassess the body form of †*O. megalodon* by addressing the question “Could it indeed have had a slender body?”. Along with the body form reassessment, we also re-evaluate its body weight, cruising speed, and ontogenetic growth parameters, which in turn offer new insights into its gigantism, life history strategies, ecology, and extinction.

## Materials and Methods

### Examined Taxa and Samples

The key specimen for this study, an incomplete vertebral specimen of †*Otodus megalodon* from the Miocene of Belgium (IRSNB P 9893; previously referred to as “IRSNB 3121”: Gottfried et al. 1996), consists of 141 associated, but disarticulated, centra up to 15.5 cm in diameter from one individual shark (Cooper et al., 2022). This specimen was found near Antwerp, but specific stratigraphic or locality information is not available (see Gottfried et al., 1996, for additional historical accounts and relevant references, including Leriche, 1926). In addition, to further reassess various biological aspects of †*O. megalodon*, this study refers to one of Bendix-Almgreen’s (1982, 1983) approximately 20 associated gigantic (as large as 23 cm in diameter) vertebrae from the Upper Miocene Gram Formation in Gram, Denmark, based on photographic evidence. Both IRSNB P 9893 and the specimen from Denmark were not associated with any teeth, but they are assumed to have come from †*O. megalodon* based on 1) their exceptionally gigantic sizes; 2) the fact that teeth of †*O. megalodon* are known from Miocene deposits at each respective area; and 3) the fact that the vertebral morphology is consistent with that of the order Lamniformes which †*O. megalodon* belongs but differs from that of another large lamniform taxon, the basking shark *Cetorhinus* (Leriche, 1926; Bendix-Almgreen, 1982, 1983; Gottfried et al., 1996; see also Shimada et al., 2021b, 2024a; Cooper et al., 2022). It should be noted that they are likely not from another large, contemporaneous, enigmatic lamniform, †*Parotodus benedenii*, known only from rarer teeth because the tooth-based maximum estimated TL for †*P. benedenii* (i.e., ca. 7.6 m by Kent, 1999; Collareta et al., 2023a) is substantially shorter than the vertebral length of 11.1 m known for IRSNB P 9893 (Figure 1B; see also Shimada et al., 2021a, table 3).

†*Otodus megalodon* belongs to †Otodontidae, but the exact systematic position of the family within Lamniformes is uncertain (Shimada, 2022; Sternes et al., 2023, 2024; Figure 1A). Therefore, we chose not to make any a priori assumption as to what lamniform taxon or taxa could have resembled the extinct shark. Furthermore, to identify lamniform-specific trends in body size and proportions, we examined taxa from a range of extant sharks in other clades. In all, our dataset comprises 145 extant species in nine orders (including Lamniformes), 38 families, and 103 genera (Appendix 1; taxonomy and classification follow Ebert et al., 2021, except *Dichichthys bigus* [Dichichthyidae], which is based on White et al., 2024). In addition, our dataset includes 20 species of Jurassic and Cretaceous (extinct) neoselachians known from complete specimens (i.e., only those fully articulated axial skeletons with preserved body outlines, particularly their head and caudal fin), including four lamniform genera, based on published illustrations of specimens (Appendix 2). These extinct taxa add one order, eight families, and 17 genera to the aforementioned taxa, resulting in a combined (extant and extinct) total dataset of ten neoselachian orders (Figure 1A) consisting of 46 families, 120 genera, and 165 species (Appendix 3).

We compiled the neurocranium length (NL; i.e., anteroposterior distance of the skull between the rostral tip and occipital centrum) data from illustrations in the literature in which scale bars and specimen orientation allowed linear calculation of NL as well as radiographic (X-ray or computed tomography) images or neurocranial specimens from non-embryonic shark individuals, each with a known TL. Specifically, we gathered data from the following papers: White (1895), Cappetta (1980), Compagno (1988, 1990), Duffin (1988), Shirai (1992), Goto (2001), Kriwet and Klug (2004), Thies and Leidner (2011), Mollen et al. (2012, 2016), Denton et al. (2018), Weigmann et al. (2020), Pfeil (2021), Vullo et al. (2021, 2024), Jambura et al. (2023), Staggl et al. (2023), Viana and Soares (2023), and White et al. (2024) (Appendices 1–2; note that incomplete fossil specimens or privately owned specimens are not included: e.g., many specimens in Pfeil, 2021). Extant individuals without TL or NL data as well as fossil specimens with missing body parts or unmeasurable TL or NL were not included. These include cases of neurocranial illustrations without scales such as those in many or all illustrations by Compagno (1990), Crawford (2014), and Villalobos-Segura et al. (2022), including occasional inadvertent omissions of scales (e.g., Shirai, 1992, plate 3A-B for *Echinorhinus brucus*; Goto, 2001, figure 13B-C for *Stegostoma tigrinum*).

The radiographically examined samples and physical neurocranial specimens used in this study are housed in the following 14 institutions: Academy of Natural Sciences of Drexel University (ANSP), Philadelphia, Pennsylvania, USA; Bernice P. Bishop Museum (BPBM), Honolulu, Hawaii, USA; Commonwealth Scientific and Industrial Research Organisation (CISRO), Hobart Tas, Australia; Field Museum of Natural History (FMNH), Chicago, Illinois, USA; Florida Museum of Natural History, University of Florida (UF), Gainesville, Florida, USA; Hokkaido University Museum (HUMZ), Sapporo, Hokkaido, Japan; Museum of Comparative Zoology (MCZ), Harvard University, Cambridge, Massachusetts, USA; National Museum of Natural History (USNM), Washington D.C., USA; Natural History Museum of Los Angeles County (LACM), Los Angeles, California, USA; Scripps Institution of Oceanography (SIO), University of California, San Diego, La Jolla, California, USA; University of Michigan Museum of Zoology (UMMZ), Ann Arbor, Michigan, USA; Yale Peabody Museum (YPM), New Haven Connecticut, USA; Zoological Museum Hamburg (ZMH), Hamburg, Germany; and Natural History Museum of Denmark (ZMUC), Copenhagen, Denmark. While there are 107 extant shark genera known to date (Ebert et al., 2021; White et al., 2024), the examined taxa account for 96.3% of all known extant shark genera. The remaining 3.7% of the genera not included in this study are: *Akheilos* (Carcharhiniformes: Scyliorhinidae), *Euprotomicroides* (Squaliformes: Dalatiidae), *Nebrius* (Orectolobiformes: Ginglymostomatidae), and *Scymnodon* (Squaliformes: Somniosidae).

### Examined Comparative Variables and Methods for TL Estimations

The units of length measurements used in this study are either centimeters (cm) or meters (m). The first main objective of this study was to determine neurocranial length (NL) and caudal fin length (CL) of †*Otodus megalodon* from the known precaudal vertebral column length, which would represent the trunk length (Figure 1B). This process includes two discrete steps. The first step (“Step 1” in Figure 1C) was to determine the neurocranial proportion (NP), trunk proportion (TP), and caudal fin proportion (CP) relative to TL, where the combination of the three “body part proportions” was considered 1 (= 100%). Based on published neurocranial illustrations or radiographic images of neurocrania of extant shark specimens with known TL, NP for each sample was calculated as NL/TL ratio (Appendix 1). For lamniforms, multiple samples of each species, if available, were measured to minimize the effects of intraspecific variation, where the average NP value for each species was calculated and used for the analysis. Ebert et al.’s (2021) illustration for each extant species was used to calculate CP by dividing CL by TL as measured on each page of the book (Appendix 3). Each TP value was obtained by first adding NP and CP and then subtracting the sum from 1 (Appendix 3). In the case of extinct taxa, NP, TP, and CP of each sample were directly determined based on “TL” as measured in each published illustration, where the average NP, TP, and CP values for each species were calculated and used for the analysis when multiple conspecific samples were available (Appendix 2). The determination of NP, TP, and CP for each species allowed the second step (“Step 2” in Figure 1C), which was to scale TP to be 100% (or “adjusted trunk proportion” [aTP] in Figure 1C) and then calculated the proportionately “adjusted neurocranial proportion” (aNP) and “adjusted caudal fin proportion” (aCP), accordingly (Appendix 3). For this study, the median aNP and aCP values were compared across taxa or operational categories as proxies to ultimately calculate the inferred NL and CL of †*O. megalodon* from the known precaudal vertebral length (Figure 1B). It should be noted that, for this specific purpose, isometric scaling of body proportions with total length is assumed by taking the median proportions (but see also the importance of allometric consideration and allometry-based analysis in the Discussion).

### Comparisons of Body Part Proportions

We addressed the question “Is it possible for †*Otodus megalodon* to have had similar body part proportions to sharks with a slender body?” to examine if there would be any possible validity to Sternes et al.’s (2024) proposition. To do so, the aNP, aTP, and aCP values for estimating the respective body part sizes of †*O. megalodon* for TL estimation (see above) were readjusted to NP, TP, and CP by considering that the combined total of the three proportional variables would add up to 1 (= 100%). These three values for †*O. megalodon* were then combined with the dataset comprising NP, TP, and CP of every examined species listed in Appendix 3 to conduct a cluster analysis using the computer software PAST (PAleontological STatistics: Hammer et al., 2001) under the Unweighted Pair Group Method with Arithmetic Means (UPGMA). This was to generate a Euclidean distance dendrogram, namely a cluster tree, to determine the taxon with the most similar body part (NP, TP, and CP) proportional relationship to our interpretation of the body part proportional relationship of †*O. megalodon*. Specifically, under similar body proportional values, we examined if †*O. megalodon* would cluster with the lamnid taxa typically used as body form proxies (e.g., Gottfried et al., 1996; Cooper et al., 2020, 2022) or another, more slender species (e.g., Sternes et al., 2024).

### Comparisons of Fineness Ratios

Once a taxon possessing a slender body with similar body part proportions with †*Otodus megalodon* was identified, the question of whether †*O. megalodon* could have had a slenderer body than lamnids was further examined from a hydrodynamic viewpoint. The body fineness ratio is a comparative index of the body slenderness sometimes used for aquatic vertebrates calculated by dividing the “body length” by the body depth, where the larger the value, the slenderer the body is (e.g., Ahlborn et al., 2009; Porter et al., 2009, 2011). Ahlborn et al. (2009) examined the fineness ratios of many cetacean taxa, and although they did not specify exactly what they meant by “body length”, it is interpreted to be the fork length that is commonly used for cetaceans. In this study, we also used the fork length and compared the body fineness ratios among sharks of interest, including previously reconstructed body forms of †*O. megalodon* as well as data from cetaceans presented by Ahlborn et al. (2009). It should be noted that Ahlborn et al.’s study (2009) did not cover some notable mysticete taxa such as the humpback and right whales. The humpback whale *(Megaptera novaeangliae)* and North Atlantic right whale *(Eubalaena glacialis)* were thus added to our dataset for comparisons and discussion of their fineness ratios as presented by Woodward et al. (2006, table 3).

The body of sharks, including †*Otodus megalodon*, is covered with dermal denticles (= placoid scales; e.g., Shimada et al., 2024a, and references therein) that are typically thrust-enhancers by reducing drag (Oeffner and Lauder, 2012), whereas whales mostly have tight smooth skin although some cetaceans have cutaneous dermal ridges or tubercles that may act to somewhat control flow (Fish and Rohr, 1999; Miklosovic et al., 2004; Fish et al., 2008; but see also Wainwright et al., 2019). However, in aquatic vertebrates (or at least in whales), the major determinant of drag is said to be body shape rather than body surface texture (Fish and Rohr, 1999), where body depth, along with large-amplitude body movements, is regarded as a major factor determining maximum acceleration (Webb, 1978). Although the magnitude of amplitude of body movements in †*O. megalodon* is uncertain, we thus consider the comparison between large sharks and whales in terms of body fineness ratios (which in turn reflects body depth) to be reasonable with the assumption that †*O. megalodon* as a gigantic marine vertebrate was likely subject to similar hydrodynamic constraints on morphology and locomotion as the similar-sized largest living whales.

The specific shark taxa and relevant references are given in the Discussion, but we note the procedure here. First, we obtained orthogonal silhouettes of the body of selected shark species from the literature. Second, using these silhouettes, we volumetrically estimated their body masses (= BW) at different total lengths for respective species, using the Paleomass R program (Motani, 2023; for further detail, see Methods for BW Estimations below). Third, we found the TL at which the volumetric body mass estimate matched the mean body mass expected for respective species based on published regression equations between body mass and fork length, and between fork length and total length. Fourth, for the remaining TL, we matched the volumetric and regression-based BW estimates by making the silhouettes more slender or stouter depending on the computation outcome by multiplying both lateral and dorsoventral body diameters uniformly across the body. Using such a factor, a likely body fineness ratio was calculated at each length for each species. The regression equations we used were based on Natanson et al.’s (2022) species-specific quantitative relationships between the fork length (their “FLOTB”) and body mass, whereas our values are based on the total length spanning from the rostral tip to the posterior tip of the caudal fin (their “TLSL”). Nevertheless, the difference is considered to have negligible effects on the overall results, because the total length and fork length are tightly correlated with each other in these sharks (Natanson et al., 2022).

### Methods for BW Estimations

The units of BW (or body mass) used in this paper are either kilograms (kg) or metric tons (t), where U.S. tons for the BW of †*Otodus megalodon* occasionally used in literature (e.g., Gottfried et al., 1996) are converted to metric tons. Our method of calculating the BW of any given shark entailed first generating a 3D computer reconstruction of the body from the outlines or silhouettes of its body in lateral and dorsoventral views based on literature (e.g., Ebert, 2014; Ebert et al., 2021; Figure 1D). Body mass was estimated from the volume of the 3D body space, but to estimate the body mass more accurately for better comparisons with reported body masses of other marine vertebrates, all non-caudal fins and non-muscular portions of the caudal fin (laterally bulged or thickened portion demarcated by a line on the lateral face of the caudal fin in published drawings) were excluded from body silhouettes (Figure 1E). Then, serial superelliptical sections were generated based on the two body silhouettes (Motani, 2023; Figure 1F). Subsequently, a 3D mesh combining all superelliptical slices was generated to allow body mass extrapolations from the volume within the 3D mesh (Figure 1G), following Motani’s (2023) methods. The calculations were made using the Paleomass package in R (R Core Team, 2024), along with the following assumptions as recommended (Motani, 2023). The mean body density was assumed to be that of the surface seawater, which is 1.027 g/cm^3^ (Stewart, 2008), which would give †*O. megalodon* near-neutral buoyancy in seawater. Although liver volume is known to exhibit positive allometry with a possible implication that “larger sharks evolved bulkier body compositions by adding lipid tissue to lean tissue rather than substituting lean for lipid tissue, particularly in the liver” (Gleiss et al., 2017, p. 1), we consider any departure from this value (1.027 g/cm^3^) to be negligibly small in free-swimming marine vertebrates for this study (see Motani, 2023). A superelliptical component range of 1.8 to 2.0 was used, following an observation that sharks tend to fit within this range (Motani, 2023). Body silhouette images were given 3,000 pixels along the body length to minimize systematic errors (Motani, 2023).

### Assessment of Cruising Speed

Cruising speeds are species-specific in sharks (Ryan et al., 2015). However, meaningful comparisons of the swimming speeds of sharks are difficult to perform due to the wide variation range in reported speeds stemming from measurements taken from individuals of different sizes or ontogenetic stages, intraspecific behavioral differences, water currents, the use of different (e.g., two-dimensional vs. three-dimensional) tracking approaches, and the duration of tracked time of each measured individual (Kai and Fujinami, 2020). In addition, reported swimming speeds of sharks also vary in distance, time unit, and/or types of speed measurements used, such as “maximum speed”, “burst speed”, and “body lengths per second”. For this study, we used published “cruising speeds” (i.e., distance per unit time: sensu Watanabe et al. 2015, which were interpreted to be equivalent to the “routine swimming speeds” of Lauder and Di Santo, 2015) as a proxy for the ordinary swimming state of each shark species. Following the approach of Shimada et al.’s (2024a) study, unit conversions were made on published cruising speeds where necessary in terms of “km per hour” (km h^-1^) to standardize our comparative speed data.

### Ontogenetic Analysis

Shimada et al. (2021b) analyzed the growth parameter of †*Otodus megalodon* using micro-computed tomography to radiographically render the incrementally deposited growth bands interpreted to have formed annually in the well-calcified body (corpus calcareum) of the vertebrae in IRSNB P 9893 (Figure 1H–J). In this present study, a new ontogenetic analysis was also conducted after the reassessment of the possible TL to emend Shimada et al.’s (2021b) growth parameter estimates. The band count of the vertebrae was 46, represented by the outermost growth band, where the largest vertebral centrum (“vertebra #4”) in the specimen measured 155 mm in diameter and was assumed to have formed when the shark was 921 cm TL and 46 years old. The 921-cm-TL estimate was based on a linear regression function describing the quantitative relationship between the maximum vertebral width and TL from 16 extant white sharks, *Carcharodon carcharias* (Gottfried et al., 1996). Each band was sequentially assigned a band number (BN), where the band at age 0 was identified by the “angle change” recognized along the inner and outer rims of the corpus calcareum (Shimada et al., 2021b; Figure 1J). Each band interval (BI) from one band to the successive band between BN 0 to BN 46, and the percent centrum radius (pCR) at each BN was calculated by treating the last BN that marked the centrum radius (CR) of 77.5 mm as 100%. Then, each extrapolated TL (eTL) from each pCR at each BN was computed by considering the estimated TL of 921 cm for the individual as 100%, and an estimated growth length (eGL) gain from one band to the next was also determined from the eTL data (Shimada et al., 2021b). The BN and eTL then formed the primary analysis using the von Bertalanffy growth function (VBGF) (von Bertalanffy, 1938) as an exploratory tool to fit the BN-TL data using the least squares method under a hypothetical supposition that each BN-TL pair (including BN 0) was obtained from a randomly sampled individual of a population even though the BN and eTL values represent dependent measurements from a single individual—a technique that has been applied to several extinct and extant elasmobranchs to elucidate their growth pattern and life history strategy (Shimada, 2008; Jacobs and Shimada, 2018; Sternes and Shimada, 2018; Shimada and Everhart, 2019; Jambura and Kriwet, 2020).

In this present study, the same BN, CR, BI, and pCR values from Shimada et al.’s (2021b) study was used to reassess the VBGF parameters for †*Otodus megalodon* using the newly extrapolated TL for IRSNB P 9893 (see Discussion). The parameters were calculated using the Desmos Inc. graphing software (www.desmos.com, v1.9.0) based on the following form of VBGF describing the length *(L)* as a function of the age of the shark *(t):*

$$L_{\left(t\right)}=L_{\infty }\left(1-e^{-k\left(t-t_{0}\right)}\right)$$

where *L*_∞_ is the estimate of asymptotic (= maximum) length, *k* the rate constant with units of reciprocal time (i.e., the time it takes for a fish in a population to reach near its mean maximum length), and *t*_0_ the theoretical time at zero length. The obtained VBGF curve allowed us to determine the body length at birth (*L*_0_) represented by its y-intercept. We also tentatively estimated the longevity of the shark using a published equation (Natanson et al., 2006) for the estimated age at 95% of *L*_∞_, i.e.:

$$\begin{array}{c} \;Longevity\;=(1/k)ln\left\{\left(L_{\infty }-L_{0}\right)/\left[L_{\infty }(1-x)\right]\right\}\\ \;\text{with}\;x=L_{\left(t\right)}/L_{\infty }=0.95. \end{array}$$

## Results

Among the extant taxa (Appendix 3), the lowest NP values are found in *Chlamydoselachus anguineus* (4.0%; Hexanchiformes: Chlamydoselachidae) followed by two of the three alopiids (5.6–5.9%; Lamniformes: *Alopias pelagicus* and *A. vulpinus*), whereas those with the highest NP values are pristiophorids (23.6–32.9%; Pristiophoriformes: *Pliotrema* spp. and *Pristiophorus* spp.) followed by *Apristurus laurussonii* (21.0%; Carcharhiniformes: Pentanchidae) and *Mitsukurina owstoni* (20.5%; Lamniformes: Mitsukurinidae). The smallest TP was found in *Alopias pelagicus* (40%), followed by *Stegostoma tigrinum* (41.4%; Orectolobiformes: Stegostomatidae) as well as in most of the taxa with the largest NP (e.g., *A. vulpinus, Apristurus, Mitsukurina, Pliotrema*, and *Pristiophorus*) besides *Alopias superciliosus* and *Pentanchus profundicolus* (Carcharhiniformes: Pentanchidae) ranging 41.6–55.0%. On the other hand, the largest TP values are found in *Squatina japonica* and *S. africana* (Squatiniformes: Squatinidae) and *Euprotomicrus bispinatus* (Squaliformes: Dalatiidae) ranging from 76.5 to 77.8%. The lowest CP values are found in *Squatina africana* and *S. japonica* (12.3 and 13.5%), followed by *Pliotrema kajae, Dichichthys bigus* (Carcharhiniformes: Dichichthyidae), and *Euprotomicrus bispinatus* (14.6–14.7%), whereas those with the highest CP values are *Alopias* spp. and *Stegostoma tigrinum* (42.8–54.1%). The median NP, TP, and CP among all the extant taxa analyzed are 12.0, 65.3, and 22.2%, respectively, and it is notable that these median values are practically identical even if all the examined extinct taxa are included (12.0, 65.4, and 22.2%, respectively: Appendix 3).

Considering only extinct and extant Lamniformes (Table 1), the lowest NP values are found in *Alopias* spp. and †*Aquilolamna milarcae* (†Aquilolamnidae) ranging 5.6–7.1%, whereas those with the highest NP values are represented by the two mitsukurinids, *Mitsukurina owstoni* and †*Scapanorhynchus lewisii*, measuring 20.5% and 17.5%, respectively. *Alopias* spp., *M. owstoni*, and †*S. lewisii* represent lamniform taxa with the lowest TP values ranging 40.0–50.7%. The TP values of the remaining lamniforms range from 58.8% *(Megachasma pelagios)* to 71.4% *(Cetorhinus maximus)*. Whereas *Alopias* spp. have the highest CP values (42.8–54.1%) among all the examined extinct and extant taxa (not only among lamniforms), lamniforms with the lowest CP values are represented by *Isurus oxyrinchus* (17.6%) and †*Ptychodus* sp. (†Ptychodontidae: 17.7%). The median NP, TP, and CP among all the extinct and extant lamniforms combined are 10.8, 63.1, and 26.2%, respectively.

If the TP values of all the examined lamniforms are considered 1 (= 100%) for standardization (“Step 2” of Figure 1C; Table 1), the lowest aNP is found in †*Aquilolamna milarcae* (10.7%), followed by *Cetorhinus maximus* (12.9%) and *Alopias* spp. (13.5–14.8%), whereas the highest aNP values are found in mitsukurinids (34.5–42.9%: *Mitsukurina* and †*Scapanorhynchus*) and odontaspidids (20.1–21.6%: *Odontaspis* spp.). The lowest aCP values among lamniforms are represented by *Isurus oxyrinchus* (17.6%) and †*Ptychodus* sp. (†Ptychodontidae: 17.7%), whereas alopiids (85.4–135.3%: *Alopias* spp.) and mitsukurininds (62.7–66.3%: *Mitsukurina* and †*Scapanorhynchus*) have the highest aCP values. The median aNP and aCP in lamniforms are 16.6 and 41.8%, respectively.

Table 2 shows the comparative aNP and aCP data by taxonomic order. The lowest and highest aNP values among all examined neoselachians are 5.8% in Hexanchiformes (specifically *Chlamydoselachus anguineus*: Appendix 3) and 65.7% in Pristiophoriformes (specifically *Pliotrema warreni*: Appendix 3), whereas the lowest and highest aCP values recorded are 16.3% in Squatiniformes (specifically *Squatina africana:* Appendix 3) and 135.3% in Lamniformes (specifically *Alopias pelagicus:* Appendix 3). By taxonomic order, the largest median aNP (53.3%) is found in pristiophoriforms with an elongated rostrum. Only one taxon of †Synechodontiformes (as well as Echinorhiniformes and †Protospinacidae) was examined in this study (see Appendix 3), and there are taxa in some other neoselachian orders with higher aNP values; however, if the single aNP is regarded as a typical (or “median”) value for the †synechodontiforms, its aNP of 26.8% represents the next highest aNP value in the “Median aNP” column. The smallest median aNP is represented by Squatiniformes (12.6%). Likewise, although only one taxon represents †Synechodontiformes (as well as Echinorhiniformes and †Protospinacidae) and there are taxa with a caudal fin that even exceeds the trunk length (e.g., *Alopias* spp.), the highest and lowest aCP values in the “Median aCP” column are also represented by †Synechodontiformes (72.0%) and Squatiniformes (17.0%), respectively. The median aNP and aCP of all the examined neoselachian species listed in Appendix 3 are 18.3% and 33.3%, respectively.

**Figure 2:**
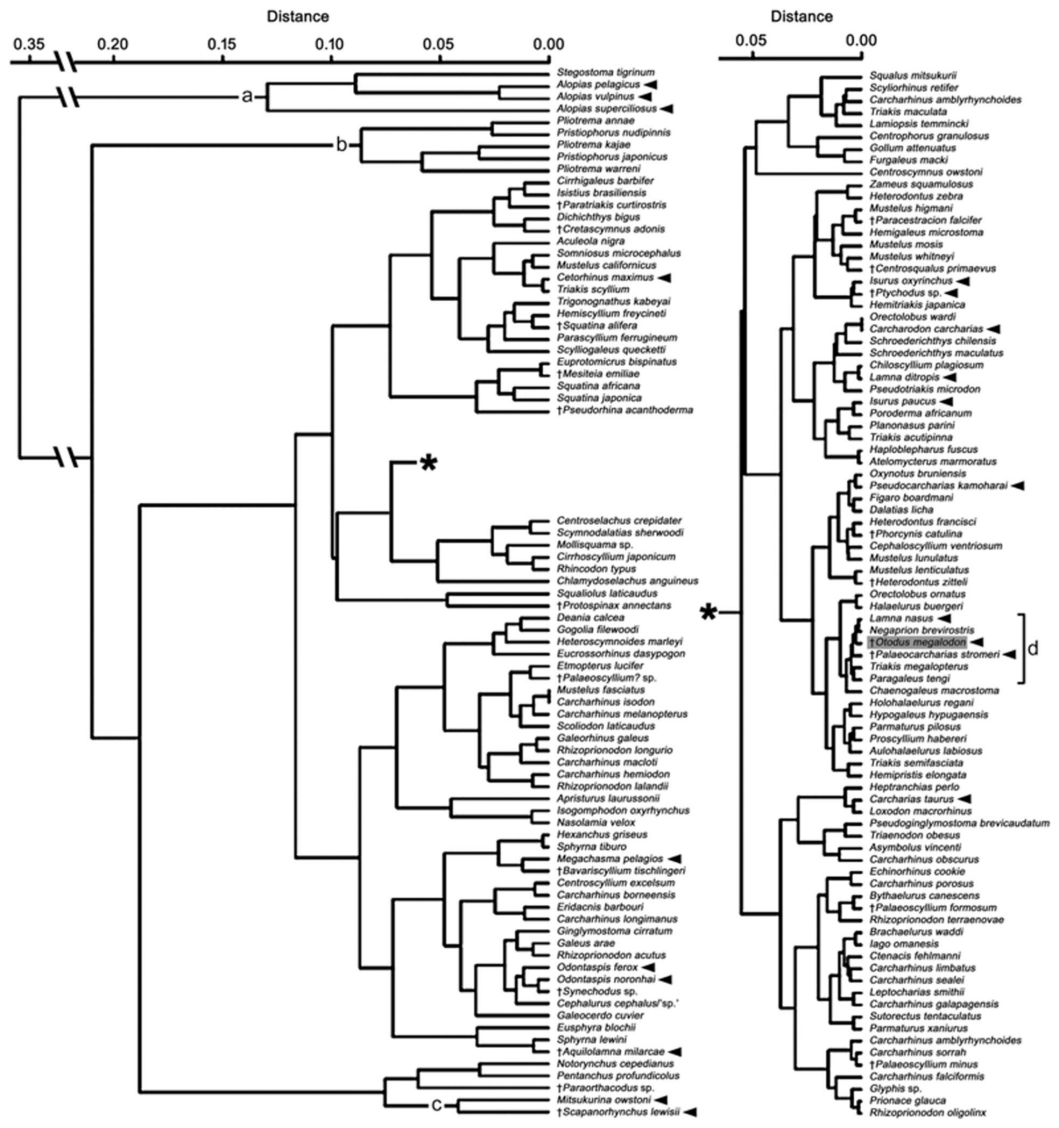
Euclidean distance dendrogram (cluster analysis) depicting the degree of difference in the relationship among the neurocranial, trunk, and caudal fin proportions across all examined taxa (NP, TP, and CP in Appendix 3), where lamniform taxa are pointed by triangle arrows and †*Otodus megalodon* is further highlighted in gray box (the cluster tree to the right is a subset of the larger tree to the left connected at the asterisk [*]). Some specific branches discussed in the text: a, alopiid lamniforms and zebra shark (Orectolobiformes: Stegostomatidae) with an exceptionally elongated caudal fin; b, pristiophoriforms with an exceptionally elongated spinous rostrum (sawsharks); c, mitsukurinid lamniforms with an exceptionally elongated non-spinous rostrum; d, selected clustering of taxa that includes †*O. megalodon*.

Figure 2 shows a cluster tree that graphically depicts the degree of differences (or similarity) in the relationship of NP, TP, and CP values across all the examined taxa (in Appendix 3), and we here note a couple of major observations. First, the most distinctive body part proportions are exhibited by Alopiidae (Lamniformes) and *Stegostoma tigrinum* (Orectolobiformes) with an exceptionally elongated caudal fin, by Pristiophoriformes with an exceptionally elongated spinous rostrum, and to a lesser extent by Mitsukurinidae (Lamniformes) with an exceptionally elongated non-spinous rostrum (“a”, “b”, and “c” in Figure 2). Second, there are no specific taxonomic, phylogenetic, ecological, or functional trends in body part proportions as exemplified by the fact that all lamniform species are largely scattered throughout the rest of the cluster tree (see distributions of arrows pointing lamniforms in Figure 2). For example, even though one branch of the tree containing †*Otodus megalodon* (“d” in Figure 2) includes two other lamniform taxa *(Lamna nasus* and †*Palaeocarcharias stromeri:* note that the lamniform attribution of †*P. stromeri* in this study is tentative: see Underwood, 2006; Landemaine et al., 2018; Jambura et al., 2019; Villalobos-Segura et al., 2023; Guinot et al., 2025), they are not only interspersed with carcharhiniforms in different families (the carcharhinid *Negaprion brevirostris*, triakid *Triakis megalopterus*, and hemigaleid *Paragaleus tengi*) but the members of the branch are also represented by diverse forms, such as the fast-cruising pelagic *Lamna nasus* with regional endothermy, the slower-cruising pelagic *Negaprion brevirostris*, demersal *Triakis megalopterus* and *Paragaleus tengi*, and even seemingly benthic †*Palaeocarcharias stromeri* (see Compagno, 1988; Duffin, 1988; Ebert et al., 2021; Shimada et al., 2024a, and references therein). Among the aforementioned taxa, *L. nasus* and *N. brevirostris* have the most similar body part proportions to †*O. megalodon* (Figure 2; see Discussion below).

## Discussion

### Total Length of †*Otodus megalodon*

Teeth and vertebrae of †*Otodus megalodon* measure up to at least 16 cm in total vertical (apicobasal) height and 23 cm in diameter, respectively (Bendix-Almgreen, 1982, 1983; Shimada, 2019), which are gigantic. Whereas TL extrapolations based on tooth sizes or vertebral diameters are now regarded to produce underestimated or otherwise unreliable TL values, particularly by comparing them with those of extant *Carcharodon carcharias* (Sternes et al., 2024), one empirical piece of information about the length of †*O. megalodon* is that an incomplete vertebral column fossil from the Miocene of Belgium putatively belonging to †*O. megalodon* (IRSNB P 9893) measures about 11.1 m when all the vertebrae are put together (Cooper et al., 2022). Although the vertebral column is considered to include a few caudal vertebrae, most of them are interpreted to represent precaudal vertebrae (Sternes et al., 2024). By assuming that the combined anteroposterior length of a few caudal vertebrae is negligible, that the vertebral column may be missing some precaudal vertebrae, and that the vertebral column was most likely slightly arched or curved in life (see various published CT scan images despite variable specimen conditions: e.g., Kim et al., 2021, figure 16; Sternes et al., 2024, figure 3), we consider 11 m to be a simplistic, reasonable, and most likely conservative precaudal vertebral column length for that specific †*O. megalodon* individual. Therefore, the lengths that are not accounted for estimating its TL are the “head” and “tail” lengths (Figure 1B).

This study shows that NP, TP, and CP values as well as aNP and aCP values vary across neoselachian sharks, where exceptionally high NP and aNP values are observed in taxa with an elongated rostrum (e.g., pristiophorids, mitsukurinids, and pentanchids, particularly *Apristurus*), and exceptionally high CP or aCP values are marked by taxa with an elongated caudal fin (e.g., alopiids). Whereas the median aNP and aCP values of all the neoselachian species examined are 18.3% and 33.3%, respectively, the median aNP and aCP values of lamniforms comprising four extinct genera and all the 15 extant species in the dataset are slightly lower (16.6%) and higher (41.8%) than those respective median values (see above; Table 2). If mitsukurinids with a uniquely elongated neurocranium and alopiids with a uniquely elongated caudal fin are excluded from the dataset of Lamniformes to minimize the effects of such uniquely extreme forms, the median aNP and aCP values are 16.6% and 32.6%, respectively (Table 2), which are comparable to, or slightly lower than, the corresponding values for all the examined neoselachian species combined. Because there is no evidence or reason presently to suggest that †*Otodus megalodon* had an exceptionally elongated rostrum or caudal fin like in the mitsukurinids and alopiids, respectively (Sternes et al., 2023), the median aNP of 16.6% and the median aCP of 32.6% attained for non-mitsukurinid/non-alopiid lamniforms are considered to serve as reasonable conservative proxies for “head” and “tail” proportions to extrapolate the TL of †*O. megalodon*, under the assumption that body proportions scale isometrically with TL (but see further discussion below).

By considering the vertebral column length of 11 m based on IRSNB P 9893 as 100%, 16.6% and 32.6% of 11 m would, respectively, yield a neurocranial length of 1.826 m and a caudal fin length of 3.586 m for that specific individual of †*Otodus megalodon*. If all three body part measurements (1.826 m + 11 m + 3.586 m) are added, the individual would have measured about 16.4 m TL. This new TL estimate is drastically larger than the previous estimate of 9.2 m TL made for the individual based on the maximum width of its largest centrum (“vertebra #4” measuring 155 mm in width) applied to a linear regression function characterizing the quantitative relationship between the maximum vertebral width and TL measurements from 16 extant white sharks (Gottfried et al., 1996).

Whereas the largest vertebra in IRSNB P 9893 measures 15.5 cm in width (or “diameter”), the largest putative vertebra of †*Otodus megalodon* on record is a specimen reported from the Miocene of Denmark (Bendix-Almgreen, 1982, 1983). According to Bendix-Almgreen (1983), the vertebra measures about 23 cm in diameter. This means that the vertebra is 1.484 times larger than the largest vertebra in IRSNB P 9893, and if this ratio is applied to the estimated TL of 16.4 m for IRSNB P 9893, the †*O. megalodon* individual from the Miocene of Denmark would have measured 24.3 m TL.

It must be noted that our 16.4-m-TL estimate for IRSNB P 9893 and the larger estimate of 24.3 m TL are hypothetical because our choice of median aNP (0.166) and aCP (0.326) values among non-mitsukurinid/non-alopiid lamniforms along with the assumption of isometry of the NP and CP with TL is arbitrary even though it is not necessarily unreasonable. Similarly, the larger estimate of 24.3 m assumes isometric scaling between TL and vertebral width through †*Otodus megalodon* ontogeny, a relationship which has not been assessed empirically. The lowest and highest aNP values in our dataset (Appendix 3) are 0.058 in *Chlamydoselachus anguineus* (Hexanchiformes: Chlamydoselachidae) and 0.657 in *Pliotrema warreni* (Pristiophoriformes: Pristiophoridae), respectively, and the lowest and highest aCP values are 0.143 in †*Pseudorhina acanthoderma* (Squatiniformes: Squatinidae) and 1.353 *Alopias pelagicus* (Lamnformes: Alopiidae). If these proportions are applied to the trunk length of IRSNB P 9893 (11 m), the neurocranial lengths and caudal lengths of that †*O. megalodon* individual are calculated to range 0.638–7.227 m and 1.573–14.883 m, respectively, which would yield the minimum and maximum possible estimates of 13.2 m TL (= 0.638 m + 11 m + 1.573 m) and 33.1 m TL (= 7.227 m + 11 m + 14.883 m), respectively. If these extreme estimates are applied to Bendix-Almgreen’s (1982, 1983) vertebral specimen, that individual could have measured as little as 19.6 m TL (= 13.2 m × 1.484) and as much as (unrealistically) 49.1 m TL (= 33.1 m × 1.484), again assuming isometry between vertebral width and TL. If we exclude taxa with an exceptionally elongated rostrum (Mitsukurinidae and Pristiophoriformes) or caudal fin (Alopiidae and Stegostomatidae) (i.e., taxa in branches “a”, “b”, and “c” in Figure 2), the largest aNP and aCP in our dataset are 0.396 in *Apristurus laurussonii* (Carcharhiniformes: Pentanchidae) and 0.720 in †*Paraorthacodus* sp. (†Synechodontiformes: †Paraorthacodontidae), respectively, which give the neurocranial and caudal fin lengths of 4.356 m and 7.920 m, respectively. If so, †*O. megalodon* represented by IRSNB P 9893 and Bendix-Almgreen’s (1982, 1983) specimen could have measured as much as 23.3 m TL (= 4.356 m + 11 m + 7.920 m) and 34.6 m TL (= 23.3 m × 1.484), respectively.

The fact that the aNP and aCP values vary significantly among neoselachians suggests that they have exploited a wide range of body part proportions through the Mesozoic–Cenozoic, including the exceptional elongation of the neurocranium or caudal fin due to unique adaptations (e.g., Alopiidae, Mitsukurinidae, and Pristiophoriformes; additional evidence for the independent evolution of elongated neurocrania in multiple neoselachian lineages is presented in Gayford et al., 2024a). Hence, we strongly recommend that all the minimum and maximum possible values given for the two specimens here should not be referenced unless their use can be justified based on empirical evidence. Rather, we regard the median aNP and aCP values to be reasonable reference points than considering the extreme values, because we do not wish to make any a priori assumptions about its body form without any direct fossil evidence beyond its unique gigantism specialization (see Shimada et al., 2021a). The remaining discussions in this paper therefore assume the 16.4-m-TL estimate for IRSNB P 9893 and the 24.3-m-TL estimate for Bendix-Almgreen’s (1982, 1983) specimen.

### Could †*Otodus megalodon* Have Had a Slender Body from a Standpoint of Body Part Proportions?

Sternes et al. (2024) suggested that †*Otodus megalodon* must have had a slenderer body than lamnids, but they did not specify exactly how slender the body was. In this study, we examined whether there are any examples of “slenderer sharks” with similar body part proportions estimated for †*O. megalodon*. For this examination, the aNP of 0.166, aTP of 1.000, and aCP of 0.326 for estimating the TL of †*O. megalodon* (see above) were readjusted to NP, TP, and CP by considering the combined total of the three body part proportions to be 1 or 100%, where the attained NP, TP, and CP of †*O. megalodon* were 0.1113, 0.6702, and 0.2185, respectively. By including these values of †*O. megalodon* to the dataset (Appendix 3), our cluster analysis indicated that *Lamna nasus* and *Negaprion brevirostris* have the most similar body part proportions to †*O. megalodon* among the taxa examined (Figure 2). It must be noted that the body part proportions of †*O. megalodon* are based on the median values of non-mitsukurinid/non-alopiid lamniforms, and thus, it is not necessarily unexpected that one of the lamniforms, *L. nasus* in this case, would be clustered close to †*O. megalodon*. However, although they belong to the same broad body form category (“Group B” sharks of Sternes and Shimada, 2020), the clustering of *L. nasus* (with a deeper body and a tall, lunate caudal fin) and *N. brevirostris* (with a rather elongate body and a highly asymmetrically caudal fin) suggests that sharks with different body plans and lifestyles may still have similar body part proportions. This observation is even more cogent when considering that *Triakis megalopterus, Paragaleus tengi*, and †*Palaeocarcharias stromeri* also closely cluster together with *L. nasus, N. brevirostris*, and †*O. megalodon* (i.e., taxa in branch “d” in Figure 2).

The clustering of long-tailed forms *(Alopias* and *Stegostoma)*, that of long-snouted extinct and extant mitsukurinids, and that of pristiophoriforms (branches “a”, “b”, and “c” in Figure 2) suggest that the proportional data do have credibility. However, the topology among the vast majority of remaining neoselachian taxa in the cluster tree shows little phylogenetic congruency (e.g., Figure 1A; Naylor et al., 2012). This is interpreted to be due to the fact that the “remaining taxa” are dominated by those with a “conventional shark design”, where the range of ways in which “100%” can be divided up into three parts (NP, TP, and CP) under the “conventional” body plan is simply limited. Regardless, one major observation that can be gleaned from Figure 2 is the scattering of lamniforms throughout the dendrogram (Figure 2), likely indicating that Lamniformes exploited wide-ranging combinations of body part proportions, which in turn seems to reflect their broad morphological and ecological diversity (e.g., Compagno, 1990; Ebert et al., 2021; Vullo et al., 2021, 2024). More importantly in the context of this present study, the close clustering of †*Otodus megalodon* with *Negaprion brevirostris* indicates that the slender body plan for †*O. megalodon* as suggested by Sternes et al. (2024) is indeed plausible.

**Figure 3:**
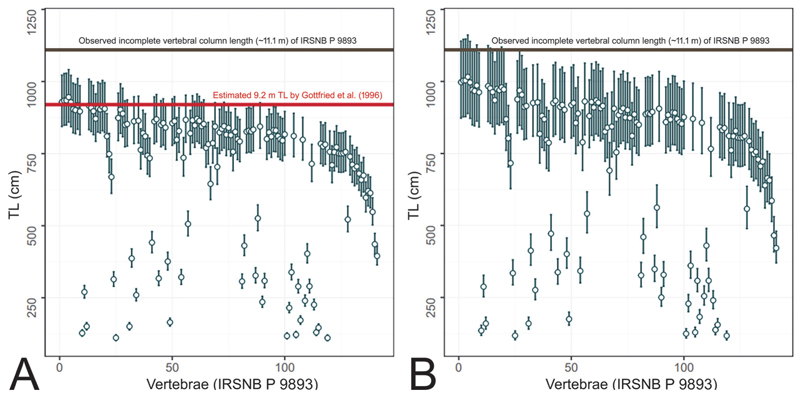
Comparisons of size estimates for each vertebra (green plots, each representing the predicted value of each centrum along with 95% prediction intervals) of IRSNB P 9893 based on the relationship between the total length (TL) and vertebral diameter in two extant lamnid sharks with the actual vertebral column length of IRSNB P 9893 reported by Cooper et al. (2022) (black horizontal line; see Figure 1B) and estimated TL of IRSNB P 9893 made by Gottfried et al. (1996) (red horizontal line). **A**, estimates based on extant white shark (*Carcharodon carcharias*) using vertebral data from Wintner and Cliff (1999; n = 111; y = 1.94 + 0.87x). **B**, estimates based on extant porbeagle shark (*Lamna nasus*) using vertebral data from Natanson et al. (2002; n = 575; y = 1.96 + 0.88x). Note that the use of these extant lamnids to extrapolate the TL of IRSNB P 9893 results in severe underestimations, including Gottfried et al.’s (1996) study.

One may ask whether †*Otodus megalodon* could have resembled *Lamna nasus* in body form, which was clustered equally close to it with *Negaprion brevirostris*. However, as Sternes et al. (2024) pointed out, a lamnid-like shark the size of †*O. megalodon* would make the vertebral column represented by IRSNB P 9893 disproportionately and unrealistically narrow (e.g., Cooper et al., 2022). Figure 3A demonstrates that TL estimates of IRSNB P 9893 based on the relationship between the TL and vertebral diameter in *Carcharodon carcharias* (e.g., Gottfried et al., 1996) do indeed yield underestimated TL compared to the actual vertebral column length of IRSNB P 9893 reported by Cooper et al. (2022) that does not even account for the head nor for much of the caudal fin. Like-wise, Figure 3B shows that the use of vertebral diameters in *L. nasus* would also result in similar TL underestimations for IRSNB P 9893, strongly suggesting that *L. nasus* too is an inappropriate comparative model for inferring the TL and body form of †*O. megalodon*. Below, we further explore the plausibility of †*O. megalodon* possessing a slenderer body compared to previous body form reconstructions (Gottfried et al., 1996; Cooper et al., 2020, 2022).

### Could †*Otodus megalodon* Have Had a Slender Body from a Hydrodynamic Standpoint?

Body size and shape affect the hydrodynamic efficiency of aquatic vertebrates, where swimming in fish-shaped vertebrates has been studied extensively (e.g., Pettigrew, 1874; Breder, 1926). Yet, most of these studies have concerned the size range of the animals typically corresponding to the order of about 10^3^ to 10^6^ in Reynolds number (Re; see Vogel, 1994). The larger size range is less well understood, although it has been pointed out that the typical body designs seen in the order of 10^3^ to 10^6^ in Re are not optimal for Re in the order of 10^7^ to 10^8^, as in large whales (Vogel, 1994). The optimum fineness ratio for minimum drag is about 4.5 (von Mises, 1945; Schlichting, 1979). Ahlborn et al. (2009) showed that a typical body fineness ratio of about 4.5 or lower was not utilized by these whales, which instead have more elongated bodies with higher fineness ratios reaching 8. They presented a numerical model suggesting that this shift in the optimal body fineness ratio with size reflects the scaling of friction and pressure drag characteristics. With the estimated maximum length between 16.4 and 24.3 m, large individuals of †*Otodus megalodon* would have had a Re in the order of 10^7^ to 10^8^ with similar constraints on optimal body fineness ratio as in whales. If so, the body design of extant lamnids with stouter bodies than whales is probably not expected in these species which in turn most likely had an elongated body.

**Figure 4:**
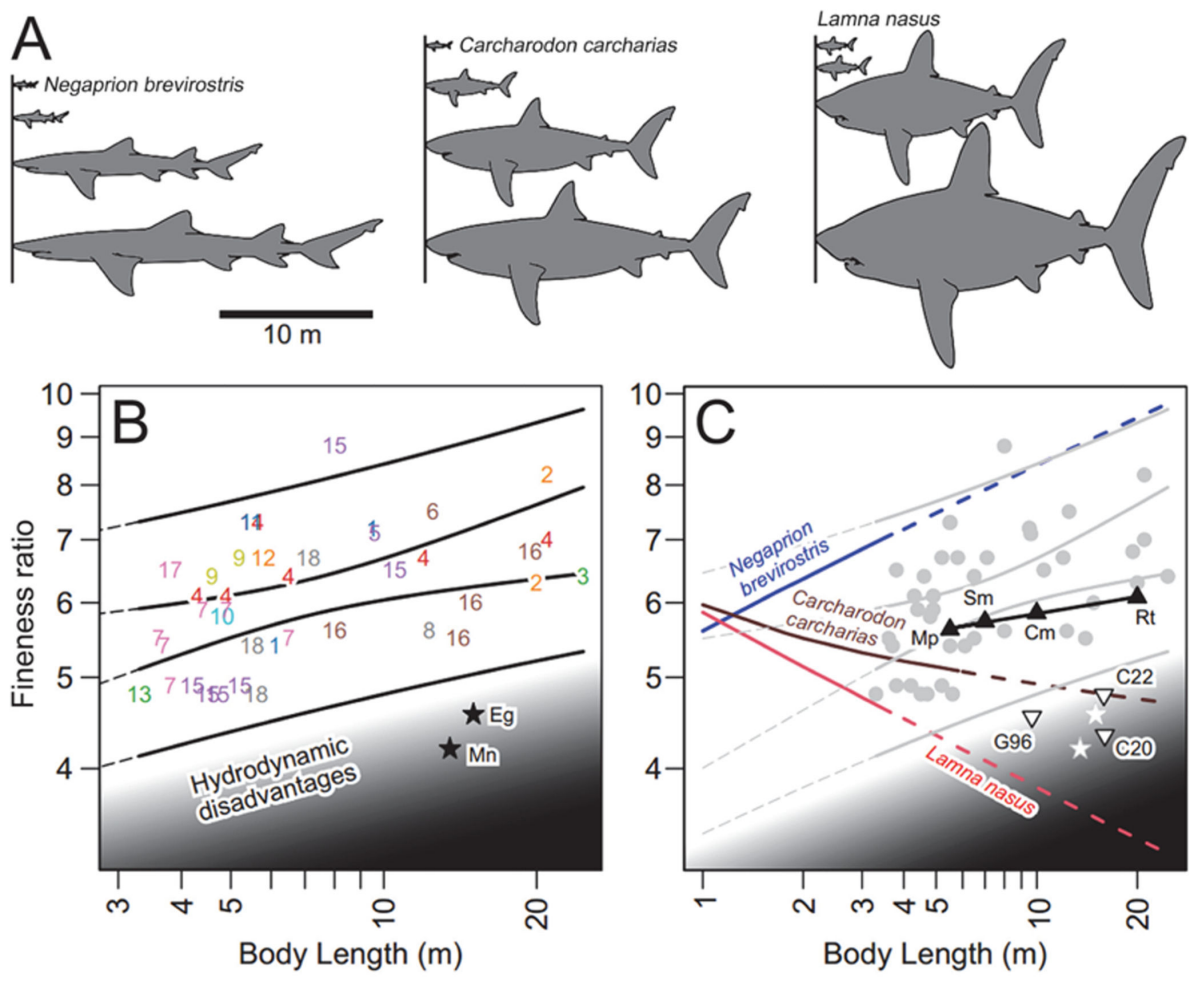
Effects of allometry (A), and comparisons of log-transformed relationship between body length and fineness ratios of cetaceans (B) and sharks of interest (C). **A**, Conceptualized effects of negative allometry in *Negaprion brevirostris* and positive allometry in *Carcharodon carcharias* (white shark) and *Lamna nasus* (porbeagle shark) if each taxon hypothetically grew to 24.3 m TL based on their species-specific relationship between body length and body weight (topmost body images based on Ebert, 2014, p. 47, 53; Ebert et al., 2021, p. 561). **B**, Body fineness ratio plotted against body length for 39 individuals of whales (numbers) based on Ahlborn et al.’s (2009, table 1) data plus two additional cetacean taxa (stars) and the region of hydrodynamic disadvantages (gray zone) (see below for species codes; narrower bands represent 95% confidence interval of the regression of the samples and wider bands 95% prediction interval for the same). **C**, Essentially same as B but with mean growth trajectories expected for the three examined extant sharks (A) (with extrapolations up to the expected body lengths of †*Otodus megalodon*: solid line, body length range known for respective species; dashed lines, extrapolated parts) as well as plots of four large extant shark taxa (solid triangles; based on Ebert et al., 2021) and three previously reconstructed †*O. megalodon* (open triangles) superimposed (see below for species and reference codes). Cetacean species codes in B: 1, *Balaenoptera acutorostrata* (common minke whale); 2, *B. borealis* (sei whale); 3, *B. musculus* (blue whale); 4, *B. physalus* (fin whale); 5, *Berardius arnuxii* (Arnoux’s beaked whale); 6, *B. bairdii* (Baird’s beaked whale); 7, *Delphinapterus leucas* (beluga whale); 8, *Eschrichtius robustus* (gray whale); 9, *Mesoplodon bidens* (Sowerby’s beaked whale); 10, *M. bowdoini* (Andrew’s beaked whale); 11, *M. densirostris* (Blainville’s beaked whale); 12, *M. ginkgodens* (ginkgo-toothed beaked whale); 13, *M. peruvianus* (pygmy beaked whale); 14, *M. stejnegeri* (Stejneger’s beaked whale); 15, *Orcinus orca* (orca); 16, *Physeter macrocephalus* (sperm whale); 17, *Pseudorca crassidens* (false killer whale); 18, *Ziphius cavirostris* (Cuvier’s beaked whale); Eg, *Eubalaena glacialis* (North Atlantic right whale); Mn, *Megaptera novaeangliae* (humpback whale). Extant shark species codes and †*O. megalodon* reference codes in C: Cm, *Cetorhinus maximus* (basking shark); Mp, *Megachasma pelagios* (megamouth shark); Rt, *Rhincodon typus* (whale shark); Sm, *Somniosus microcephalus* (Greenland shark); C20, †*O. megalodon* at 16 m TL by Cooper et al. (2020, figure 2d); C22, †*O. megalodon* at 15.9 m TL by Cooper et al. (2022, figure 1N); G96, †*O. megalodon* at 11 m TL by Gottfried et al. (1996, figure 7).

Based on the regression line for each species presented by Natanson et al. (2022, tables 16A, 20A, 20B, 31A), we tested if any of the sharks of interest—namely, the carcharhinid *Negaprion brevirostris* as well as two lamnids, *Lamna nasus* and *Carcharodon carcharias*—had a mean growth trajectory that results in such an elongated body at very large sizes. Orthogonal silhouettes (e.g., Figure 1D, 1E) of each species were generated to estimate the volumetric mass (Figure 1F, 1G), and the calculated volumetric mass was fitted to the regression-based mass estimate at different lengths for each species. If larger individuals had volumetric estimates that were larger than regression-based estimates, it was determined that the silhouettes would be too stout for these larger sizes, and the opposite was true for smaller individuals. The results are that the three species exhibit strong allometric relationships between body mass and length, suggesting that their body fineness ratio changes with growth, either positively or negatively depending on the species. In the case of *N. brevirostris*, the theoretical individuals that were experimentally fit to the regression line between the total length and body weight (TL-BW) become slender with the increase in fineness ratios because BW exhibits negative allometry as TL increases (Figure 4A). On the other hand, in *L. nasus* and *C. carcharias*, the fineness ratio decreases as TL increases because BW exhibits positive allometry as TL increases (Figure 4A; see also Hunt et al., 2024). We should note that, even if the original silhouettes used are slightly inaccurate compared to the outlines of the actual sharks, they will not affect the overall observed allometric trend of each species.

Figure 4B shows the fineness ratio plotted against body length for 41 individuals of whales consisting of 20 species in 11 genera based on Ahlborn et al.’s (2009, Table 1) study combined with two additional taxa that were not included in their work (see Materials and Methods). The plots show that large migratory cetaceans tend to have a fineness ratio as high as 8, where larger body mass is compensated for to continue providing minimum drag helping to reduce the energy cost of swimming (Ahlborn et al. 2009). An 8-m-long individual of *Orcinus orca* with an unusually slender body for the species is a clear outlier (Ahlborn et al. 2009), but it would not affect the overall trend seen in the remaining taxa. The gray region in the graph represents the area mostly not utilized by the whales due to hydrodynamic disadvantages, which was implied by Ahlborn et al.’s (2009) study. Although body fineness ratios can approach as low as about 4, such as for *Eubalaena glacialis* and *Megaptera novaeangliae*, the graph illustrates that those cetaceans with stout bodies would come with hydrodynamic disadvantages. Based on this graph, we then addressed the question of where in the graph the following three sets of sharks of interest would fall: 1) *Negaprion brevirostris, Lamna nasus*, and *Carcharodon carcharias* that are artificially enlarged to the size of †*Otodus megalodon* under their respective allometric growth (Figure 4A); 2) previous reconstructions of †*O. megalodon* (Gottfried et al., 1996; Cooper et al., 2020, 2022); and 3) the largest extant sharks (*Cetorhinus maximus* [basking shark: Lamniformes], *Megachasma pelagios* [megamouth shark: Lamniformes], *Rhincodon typus* [whale shark: Orectolobiformes], and *Somniosus microcephalus* [Greenland shark: Squaliformes]). Figure 4C shows the same cetacean plots as in Figure 4B, but these sets of sharks of interest are added.

The relationships between the fineness ratios and body lengths of the three extant shark taxa *(Negaprion brevirostris, Lamna nasus*, and *Carcharodon carcharias:* Figure 4A) in Figure 4C reveal two major contrasting trends. The trajectory of *N. brevirostris* continues parallel to the general plot distribution of whales, whereas the trajectories of both *L. nasus* and *C. carcharias* extend to the region of “hydrodynamic disadvantages”. Although the exact TL-BW relationship of †*Otodus megalodon* is uncertain, Figure 4C highlights the importance of the consideration of possible allometry when assessing the body form of large extinct organisms like †*O. megalodon* (see also Gayford et al., 2024b). For instance, it clearly demonstrates that positive allometry leads to an excessively stout body (e.g., Figure 4A), which would increase drag and reduce hydrodynamic efficiency. Whereas there is a general size limit of 7 m TL for non-plank-tivorous sharks with †*O. megalodon* representing an outlier (Pimiento et al, 2019; Shimada et al., 2021a), more significantly, Figure 4C appears to suggest that *L. nasus* and *C. carcharias* larger than 3.7 and 6.4 m TL, respectively (i.e., their maximum known length: Ebert et al., 2021), would lead to a body too stocky to account for energetically sustainable swimming. It must be emphasized that these allometric curves (represented by broken lines in Figure 4C) are extrapolations, and the reality is that there is no reason to assume the gradient of these allometric curves will remain consistent through ontogeny where data do not exist. Nevertheless, perhaps the most profound implication of Figure 4C is that sharks showing negative allometry (e.g., *N. brevirostris*) can potentially achieve gigantism while maintaining hydrodynamic efficiency, whereas those with strong positive allometry (e.g., *L. nasus*) are doomed to become inefficient swimmers if their size reaches the range of the hydrodynamically disadvantageous zone unless the allometric scaling coefficient changes through ontogeny to avoid it.

Figure 4C also shows the plots representing the relationships between the fineness ratio and body length for the three previous reconstructions of †*Otodus megalodon* (Gottfried et al., 1996; Cooper et al., 2020, 2022). All three previous works relied on or made an a priori assumption that the biology of †*O. megalodon*, including its body form, must have been similar to that of extant *Carcharodon carcharias*. While Gottfried et al. (1996) simply speculated †*O. megalodon* to have had a slightly more massive body than the extant *C. carcharias*, Cooper et al. (2020, 2022) incorporated other extant lamnid taxa to reconstruct †*O. megalodon*, which resulted in their reconstructions being slightly stockier than the extant *C. carcharias* (however, it should be noted that the exact procedure of lamnid-based “model adjustments” is not described adequately in either paper by Cooper et al.). The artificial increase in stoutness in all three studies may mimic the effects of positive allometry seen in *Lamna nasus* and *C. carcharias* (e.g., Figure 4A); thus, it is not surprising that the three †*O. megalodon* plots occur on or between the trajectory lines of the two extant lamnids (Figure 4C). The fact that they are closely clustered with the plots of extant *Eubalaena glacialis* and *Megaptera novaeangliae*, which in turn comprise some of the stockiest-bodied large cetaceans, implies that the body forms represented by the three †*O. megalodon* reconstructions are theoretically viable. However, because the three †*O. megalodon* plots occur within the hydrodynamic disadvantageous region, our findings based on Figure 4C do not support Cooper et al.’s (2022) contention that †*O. megalodon* resembled *C. carcharias* or stocky lamnids in body form and was a fast or hydrodynamically efficient swimmer at the same time.

Figure 4C additionally shows the plots of the following four largest non-alopiid extant shark taxa at their maximum known lengths *(Cetorhinus maximus, Megachasma pelagios, Rhincodon typus*, and *Somniosus microcephalus*: see Ebert et al., 2021; Shimada et al., 2021a). Not only do the four extant shark taxa have relatively high fineness ratios (>5) and occur within the plot distribution of the vast majority of cetaceans, but also their plots are situated between the trajectory line of *Negaprion brevirostris* and the lines of the two extant lamnids *(Lamna nasus* and *Carcharodon carcharias)* as well as outside of the hydrodynamically disadvantageous region. Effectively, this finding indicates the interpretation that †*Otodus megalodon* could have had a more elongated body compared to the extant *C. carcharias* and other lamnids (Sternes et al., 2024) is quite plausible. More significantly, the four plots line up linearly almost perfectly, suggesting that pathways to gigantism are conserved across phylogenetically disparate neoselachians at least based on the present data. If this interpretation is taken at face value, †*O. megalodon* at 16.4 m and 24.3 m TL would have had a fineness ratio of 6.01 and 6.15, respectively, based on the linear relationship through the four plots in Figure 4C.

**Figure 5:**
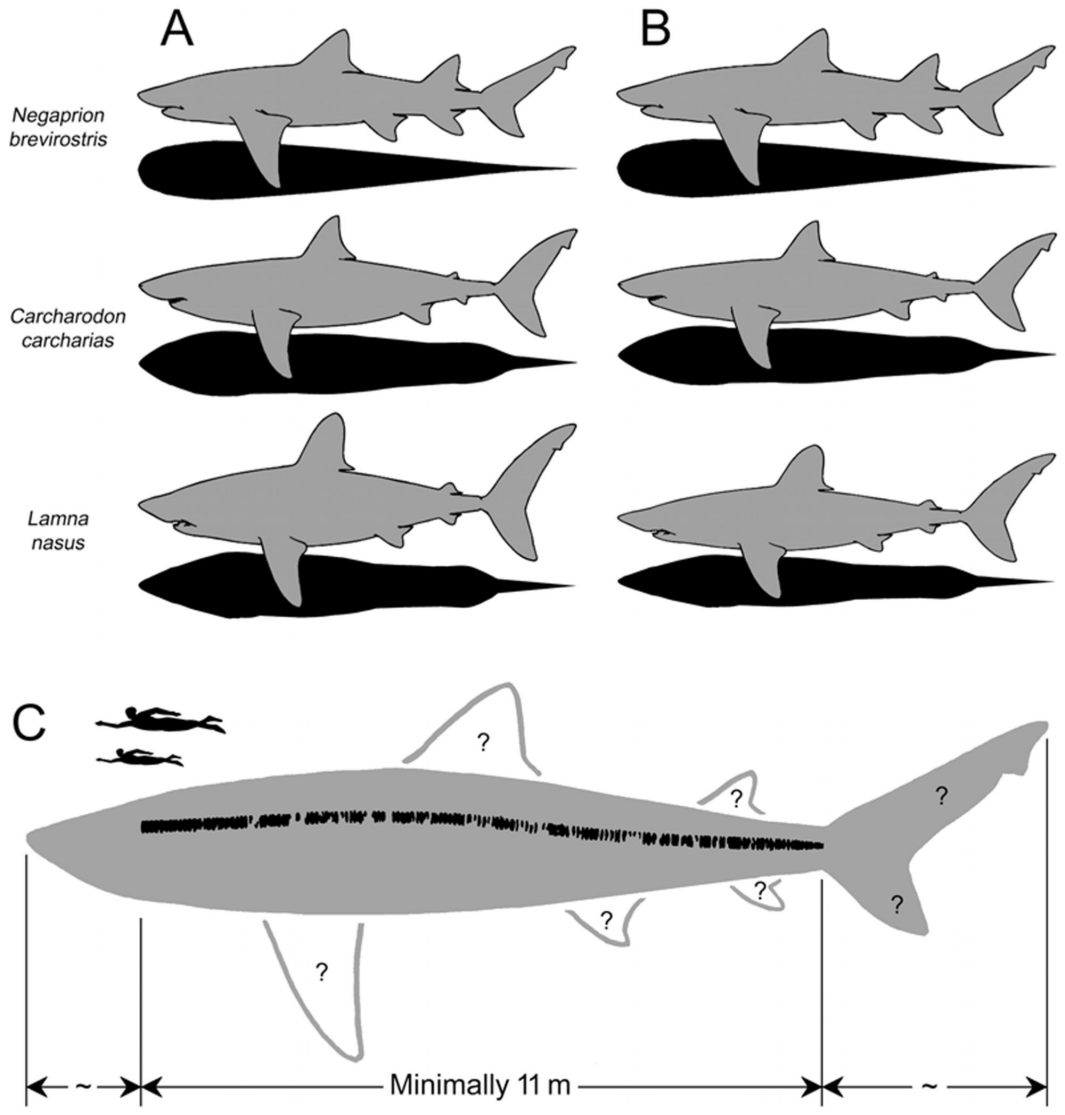
Silhouettes (not to scale) of *Negaprion brevirostris* (lemon shark), *Carcharodon carcharias* (white shark) and *Lamna nasus* (porbeagle shark) in lateral (gray) and dorsoventral (black) views (A), their hypothetically manipulated shapes (B), and highly tentative, conceptualized reconstruction of †*Otodus megalodon* (C). **A**, original morphology based on Ebert (2014, p. 47, 53) and Ebert et al. (2021, p. 561; see also text). **B**, hypothetical morphology of each shark in A after forcefully applying fineness ratio of 6.15, which is the mean fineness ratio obtained from the regression line for the four large extant sharks (solid triangles) in Figure 5G. **C**, Highly tentative reconstruction of †*O. megalodon* with a fineness ratio of approximately 6.08 onto which a silhouette of the reconstructed vertebral column of †*O. megalodon* by Cooper et al. (2022) (IRSNB P 9893: Figure 1B) has been superimposed with slight curvatures added. Two silhouettes of *Homo sapiens* (swimmers; from Jambura and Kriwet, 2020, figure 4) of different sizes illustrate the relative length of the reconstructed †*O. megalodon* at 16.4 m TL (top swimmer) and 24.3 m TL (bottom swimmer) for comparisons, but it must be emphasized that *H. sapiens* and †*O. megalodon* never coexisted. Tildes (∼) denote inferred proportions that remain to be tested through the discovery of a well-preserved complete skeleton; also note that the size and shape of all the fins, including the caudal fin, remain highly hypothetical (see text for detail).

Figure 5A shows the body form of *Negaprion brevirostris, Carcharodon carcharias*, and *Lamna nasus* (left half), and how they would appear if a fineness ratio of 6.15 is hypothetically applied (right half). It visually shows that the body form of *N. brevirostris* requires the least amount of morphometric transformation among the three species as it already has a fineness ratio of about 6. It coincidently implies that the general body form of †*Otodus megalodon* could have indeed somewhat resembled that of *N. brevirostris* (e.g., Figure 2), even though the fineness ratio of approximately 6 for †*O. megalodon* was derived completely independently from the trendline running through the four large extant taxa *(Cetorhinus maximus, Megachasma pelagios, Rhincodon typus*, and *Somniosus microcephalus:* Figure 4C). Based on this result, Figure 5C illustrates a highly tentative, conceptualized reconstruction of †*O. megalodon* with a fineness ratio of about 6.08 (i.e., about halfway between 6.01 and 6.15 calculated), respectively, for 16.4-m-TL and 24.3-m-TL †*O. megalodon* (see above). It is superimposed with a silhouette of IRSNB P 9893 (Figure 1B), with a slight curvature added based on published CT images of extant shark skeletons, including *N. brevirostris* (e.g., McQuiston et al., 2017, figure 1; Sternes et al., 2024, figure 3). Although the position of fins in sharks is known to be relatively consistent across taxa (Thomson and Simanek, 1977), the exact sizes and shapes of all the fins of †*O. megalodon* remain uncertain. It must be emphasized that the NP and CP relative to the trunk proportion remain inferential based on this present study. In addition, this reconstruction (and this entire study for this matter) assumes that IRSNB P 9893 (Figure 1B) consists of a complete set of precaudal vertebrae. If more precaudal vertebrae were originally present in the individual, it would mean that the body was likely even more elongated than depicted in Figure 5C assuming that the neurocrania length and caudal fin length used remain consistent.

It must also be emphasized that practically every aspect of the reconstructed body form of †*Otodus megalodon* in Figure 5C remains uncertain, and the discovery of a well-preserved complete skeleton of †*O. megalodon* in the fossil record is still needed to decipher its exact body form. Although there is some evidence that intra-specific variation in morphology, including in the neurocranium, does not obscure interspecific differences (de Oliveira Lana et al., 2021), it should also be noted that most of the taxa listed in Appendix 3 do not account for possible intraspecific variations (e.g., individual, sexual, and ontogenetic differences) in body part proportions even if they are present, where the three proportional values (NP, TP, and CP) with a sum of 1 or 100% are inter-dependent from one another as a change in one value affects the other two values. Nevertheless, also from the hydrodynamic standpoint, our study strongly indicates Sternes et al.’s (2024) interpretation that “†*O. megalodon* was more elongated than *C. carcharias*” is more parsimonious than the previous view that †*O. megalodon* resembled closely *C. carcharias* (Gottfried et al., 1996; Cooper et al., 2020, 2022). In fact, our findings confirm that TL extrapolations based on dental or vertebral measurements from extant *C. carcharias* (e.g., Randall, 1973; Shimada, 2003, 2019; Pimiento et al., 2010; Pimiento and Balk, 2015; Reolid and Molina, 2015; Razak and Kocsis, 2018; Herraiz et al., 2020; Perez et al., 2021) yield underestimated values (Sternes et al., 2024).

Whereas the origin of the genus †*Otodus* can be traced back to the Danian (lower Paleocene), it is worth noting that the tendency towards gigantism in the †*Otodus* clade began with the evolution of the geologically oldest chronospecies, †*O. obliquus*, as exemplified by the fact that its anterior teeth attained as tall as 9 cm in total tooth height by the Ypresian (Early Eocene: Cappetta, 2012). Even though this is too likely underestimated as the TL extrapolation relied on the relationship between the tooth size and TL of extant macrophagous lamniforms, including *Carcharodon carcharias*, †*O. obliquus* is thought to have reached at least 8 m TL (Shimada et al., 2021a). The subsequent chronospecies further increased their overall tooth size, developed serrations, and reduced the size of lateral cusplets, and they are generally understood to consist of: †*O. auriculatus* in the Late Eocene; †*O. angustidens* in the Oligocene; †*O. chubutensis* in the Early to mid-Miocene; and ultimately †*O. megalodon* in the mid-Miocene through Early Pliocene (e.g., Pimiento and Balk, 2015; Trif et al., 2016; Perez et al., 2019; Ballell and Ferrón, 2021; Shimada et al., 2021a). If Shimada et al.’s (2024a) inferences about the range of the maximum TL of the chronospecies in-between †*O. obliquus* and †*O. megalodon* are applied, they (i.e., †*O. auriculatus*, †*O. angustidens*, and †*O. chubutensis*) must have minimally attained the body size of †*O. obliquus* (at least 8 m TL: Shimada et al., 2024a) without reaching that of †*O. megalodon* (24.3 m TL: this study). Regardless of their exact maximum attainable body sizes, it seems clear that all members of the †*Otodus* clade consistently overcame the general size limit of 7 m TL recognized for non-planktivorous sharks (Pimiento et al., 2019; Shimada et al., 2021a) by the Early Eocene. The trend of gigantism in †*Otodus* also corresponds well with the elevated tropic position through the clade that began with †*O. obliquus* (Kast et al., 2022).

### Body Weight of †*Otodus megalodon*

Most prominent previous BW estimates of †*Otodus megalodon* are those presented by Gottfried et al. (1996, table 1) and Cooper et al. (2022, table 1). Gottfried et al.’s (1996, table 1) BW estimates included those of the “largest fetus”, “smallest neonate”, largest immature male and female, smallest mature male and female, “largest mature female”, and “large male”, where the smallest and largest estimated BW values were 430 kg (0.4 t) for the “smallest neonate” (3.6 m TL) and 103,197 kg (103 t) for the “largest mature female?” (20.3 m TL). On the other hand, Cooper et al. (2022) gave the BW of 61,560 kg (62 t) at 15.9 m TL. However, the validity of these previous estimates (Gottfried et al., 1996; Cooper et al., 2022) is questionable, because they were based on the a priori assumption that †*O. megalodon* resembled the extant *Carcharodon carcharias* or lamnids (see above).

Although we tentatively infer †*Otodus megalodon* to have had a fineness ratio of about 6.01–6.15 (Figure 5C), the exact BW remains difficult to decipher because of many uncertainties and assumptions (see above). Nevertheless, the hypothetical *Negaprion brevirostris, Carcharodon carcharias*, and *Lamna nasus* with a fineness ratio of 6.01 and a TL of 16.4 m would have weighed about 30.3 t, 33.7 t, and 26.9 t, respectively (average of roughly 30 t), while those with a fineness ratio of 6.15 (Figure 5B, left half) and a TL of 24.3 m would have weighed about 94.2 t, 104.7 t, and 83.6 t, respectively (average of roughly 94 t). Therefore, the individual with an estimated TL of 16.4 m represented by IRSNB P 9893 could have weighed about 30 t and the currently largest estimated †*O. megalodon* (24.3 m TL) about 94 t. These estimates are roughly comparable with those of extant blue whales *(Balaenoptera musculus)* measuring 17 m (37 t) and 25 m (119 t) in fork length, respectively (Motani and Pyenson, 2024, table 1; see also Paul and Larramendi, 2025) as well as the largest extant whale shark *(Rhincodon typus)* measuring up to about 18.8 m TL and weighing close to 34 t (McClain et al., 2015, and references therein).

### Cruising Speed of †*Otodus megalodon*

†*Otodus megalodon* was traditionally characterized as a fast-swimming shark, with previously estimated cruising speeds ranging from 4.8 to 5.1 km h^-1^ (Jacoby et al., 2016; Ferrón, 2017; Cooper et al., 2022). However, it was reinterpreted to be a slower-cruising shark (1–3 km h^-1^) compared to extant lamnids overall (from the slowest *Carcharodon carcharias* of 3.2 km h^-1^ to the fastest shortfin mako *[Isurus oxyrinchus]* of 6.7 km h^-1^) (Shimada et al., 2024a) on the basis of the morphological property of its placoid scales, specifically interkeel distances maintained through ontogeny in sharks with keeled scales (see Raschi and Elsom, 1986). Subsequently, Sayama et al. (2024) examined the keel properties of placoid scales sampled from various body positions of a 3.2-m-TL *C. carcharias* and mathematically arrived at relatively similar “migration speeds” (which is interpreted to be equivalent to cruising speed) for *C. carcharias* (8.3 km h^-1^) and †*O. megalodon* (9.7 km h^-1^), which were drastically slower than that of *I. oxyrinchus* (17.7 km h^-1^). It should be pointed out that Sayama et al.’s (2024) calculations assumed the estimate of 11.7 m TL for the †*O. megalodon* individual based on *C. carcharias*, and if their method is used, a 24.3-m-TL †*O. megalodon* would have had a migration speed of 10.8 km h^-1^. However, speed estimates based on Sayama et al.’s (2024) theoretical method appear to generate overestimations, considering that the mean of the mean observed cruising speeds of *C. carcharias* and *I. oxyrinchus* are only 3.5 km h^-1^ (range of 1.7–8.1 km h^-1^; n = 38) and 3.3 km h^-1^ (range of 1.0–6.7 km h^-1^; n = 26), respectively, based on data from numerous studies compiled by Cooper et al. (2022, data S1; but also note potential problems with Cooper et al.’s dataset as discussed by Shimada et al., 2024a). Because the reported cruising speeds of *Lamna* spp. (3.6–3.9 km h^-1^: Cooper et al., 2022, data S1) are also faster than reported for *C. carcharias*, therefore, even if the estimated cruising speed of †*O. megalodon* could have reached a level comparable with *C. carcharias* (Sayama et al., 2024), it would still have been an overall slower swimmer than lamnids as a whole based on the presently available cruising speed data (Cooper et al., 2022, data S1; Shimada et al., 2024a, table 1).

*Rhincodon* and *Cetorhinus* are the two largest extant sharks or fishes (Ebert et al., 2021; Figure 4C) and have cruising speeds that are much faster (3.1–3.9 km h^-1^) than the other two sharks, *Megachasma* and *Somniosus* (1.3–1.5 km h^-1^) (table 3). The ability of *Rhincodon* and *Cetorhinus* to achieve relatively high cruising speeds is interpreted to be due to their planktivory near the sea surface where larger spatial coverages are needed for filter-feeding (Shimada et al., 2024a). The lack of any modern examples of macrophagous (= non-planktivorous) sharks that reach or exceed the sizes of the two aforementioned planktivores complicates the inferences about the cruising speed of †*Otodus megalodon*. Unlike *Rhincodon*, which is ectothermic and *Cetorhinus* which is inferentially endothermic (e.g., see Dolton et al., 2023), †*O. megalodon* was endothermic based on geochemical evidence (Griffiths et al., 2023). However, its warm-bloodedness is interpreted to have been possibly used largely for facilitating digestion compared to promoting fast-swimming like lamnids because its placoid scales suggested its cruising to be overall slower than the extant lamnids collectively (Shimada et al., 2024a; see above). Whereas the cosmopolitan occurrences of †*O. megalodon* fossils even to include deep oceans far from continental shorelines indicate that †*O. megalodon* was capable of long-distance swimming, whether the wide geographic distribution was due to a migratory behavior (e.g., Pollerspöck et al., 2023, and references therein) cannot be ascertained decisively from the fossil record. However, if †*O. megalodon* was capable of at least cruising as fast as the slowest lamnid, *Carcharodon carcharias* (Sayama et al., 2024; see above), it is possible that †*O. megalodon* could have exhibited a migrating behavior like the extant *C. carcharias* (e.g., Skomal et al., 2017, and references therein), which has an average cruising speed (3.5 km h^-1^; see above) comparable to *Rhincodon* and *Cetorhinus*.

The three largest cetacean taxa in Figure 4C are *Balaenoptera borealis* (sei whale), *B. musculus* (blue whale), and *B. physalus* (fin whale) (“2”–”4” in Figure 4B). These are all migratory baleen whales that filter feed near the sea surface (Mizroch et al., 1984a, 1984b, 1984c) with mean cruising speeds ranging between 7.9 and 10.4 km h^-1^ (table 3). The next largest cetacean in Figure 4C is *Physeter macrocephalus* (sperm whale), a toothed whale that actively hunts for prey in deep waters (e.g., Aoki et al., 2012) with a mean cruising speed of 6.4 km h^-1^ (table 3). On the other hand, the other two cetaceans in Figure 4C, *Eubalaena glacialis* (North Atlantic right whale) and *Megaptera novaeangliae* (hump-back whale), have stocky bodies and are in the region of hydrodynamic disadvantages, and they indeed record slower mean cruising speeds than at least the three *Balaenoptera* taxa (table 3). Whereas *E. glacialis* is particularly slow (1.9 km h^-1^), however, *M. novaeangliae* is considerably fast (7.5 km h^-1^) (table 3). This could be explained by the fact that, while *M. novaeangliae* is quite migratory, its pectoral fins are highly enlarged for its unique maneuvering behavior during feeding (Hain et al., 1982), which would require a stocky build to reinforce the rigidity of the body trunk. Nevertheless, it is important to note that the cruising speeds of the gigantic whales outside of the hydrodynamically disadvantageous region are much faster than those of the two large planktivorous sharks *(Rhincodon* and *Cetorhinus)* and even lamnids (see above). This fact could be simply due to the difference in swimming modes between sharks (lateral strokes) and aquatic mammals (vertical strokes) as well as to the fact that the skeleton of sharks is cartilaginous whereas that of whales is osseous. Therefore, the use of cruising speeds observed in whales is interpreted to be inappropriate for inferring the cruising speed of †*O. megalodon*.

Our allometric analysis suggests that it is unlikely that †*Otodus megalodon* could have been stout and gigantic at the same time because such a body form would have imposed excessive energetic costs for swimming (Figure 4C; see above). The fact that the known size ranges of both *Carcharodon carcharias* and *Lamna nasus* do not extend to the region of hydrodynamic disadvantages in Figure 4C suggests that the graph appears credible. Whereas *Megaptera novaeangliae* could achieve relatively high cruising speeds despite its stocky body (see above), sharks are less likely to be able to defy such hydrodynamic constraints because of their cartilaginous skeleton. It is noteworthy that the range of interkeel distances of placoid scales in †*O. megalodon* completely overlaps with the common interkeel distance in *Negaprion brevirostris* that has a typical cruising speed of 2.1 km h^-1^ (based on the middle number of “0.44–0.71 m s^−1^” reported by Sundström and Gruber, 1998; see Shimada et al., 2024a) or a mean of the mean cruising speeds of 2.4 km h^-1^ (range of 2.1–2.7 km h^-1^: Cooper et al., 2022, data S1). Even if †*O. megalodon* was able to cruise as fast as the slowest lamnid, *C. carcharias* (Sayama et al., 2024; see above), there is currently no empirical evidence to support that †*O. megalodon* had a typical cruising speed as high as 4.8–5.1 km h^-1^ (e.g., Jacoby et al., 2016; Ferrón, 2017; Cooper et al., 2022). Rather, it seems reasonable to assert from the presently available data that the typical cruising speed of †*O. megalodon* ranged around 2.1–3.5 km h^-1^. If so, †*O. megalodon* would have typically needed to resort to burst swimming for prey capture (Shimada et al., 2024a). Sayama et al.’s (2024) placoid scale-based theoretical model suggested the “hunting speed” of †*O. megalodon* to be about 21 km h^-1^; however, because of the discrepancies between the estimated and observed cruising speeds of *C. carcharias* in their study (see above), the validity of their various speed estimates remains questionable. In our view, estimating the maximum burst or hunting speed of †*O. megalodon* would be too speculative at least at the present time. However, regardless of its exact cruising speed, we contend that the cruising speed of †*O. megalodon* possibly stayed relatively constant throughout ontogeny, considering that inter-keel distances of placoid scales indicative of relative swimming speeds in sharks remain relatively constant throughout their life (Raschi and Elsom, 1986). If so, as for cetaceans (Ahlborn et al., 2009), larger body mass could have been compensated for by increasing the fineness ratio as †*O. megalodon* grew larger, which concomitantly helped maintain minimum drag to continuously reduce the energy cost of swimming. If this is indeed the case, it also implies that the body form of †*O. megalodon* underwent at least some degree of allometric changes through ontogeny, and it is in the realm of possibility that the body growth pattern could have followed the allometric trend seen across the four large extant shark taxa *(Megachasma, Somniosus, Cetorhinus*, and *Rhincodon)* in Figure 4C.

### Growth Model and Life History Strategies of †*Otodus megalodon*

**Figure 6:**
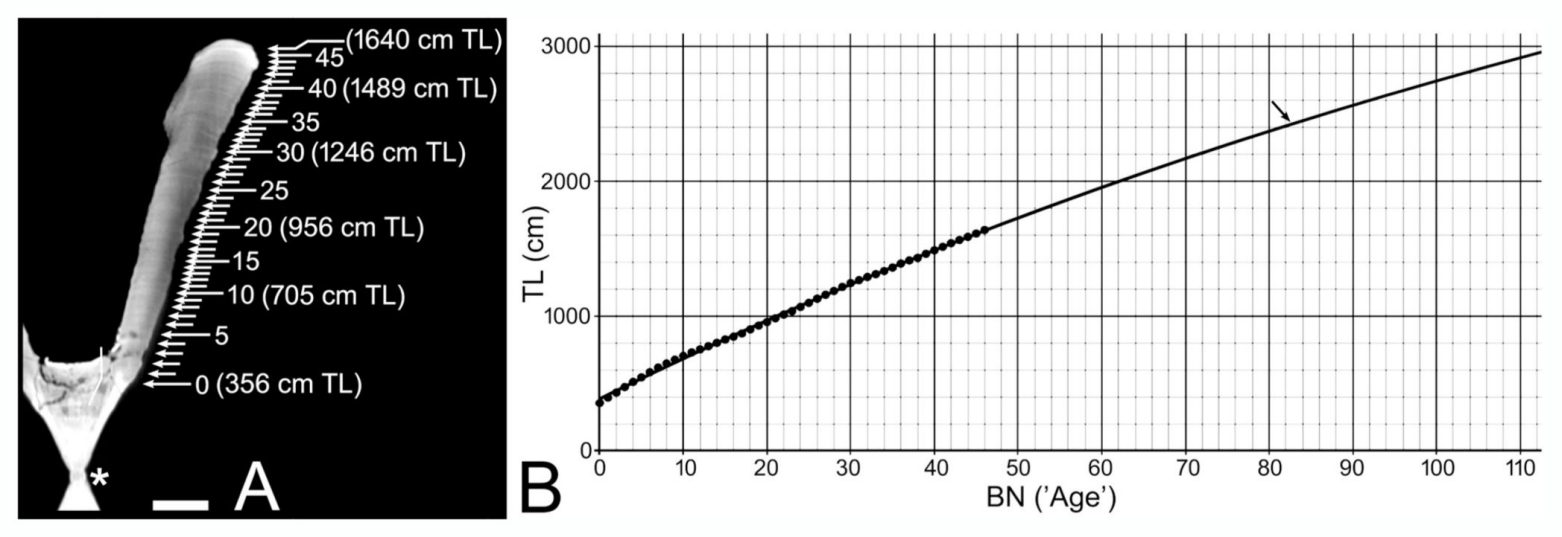
Ontogenetic growth analysis of †*Otodus megalodon* based on IRSNB P 9893 (see text for detail). **A**, Computed tomographic image of a sagittal cross-sectional view of the largest vertebrae (“centrum #4”) in IRSNB P 9893 showing incremental growth bands presumably formed annually along with estimated total length (TL) shown at every 10 growth band (cf. Figure 1J; scale bar equals 1 cm; * = center of vertebra; bent line = “angle of change”: see Shimada et al., 2021b). **B**, von Bertalanffy growth function (VBGF) fitted to data points (Table 4) that shows the relationship between growth band numbers (BN, or “age” of the individual in years) and TL (circles indicate plots based on IRSNB P 9893; arrow points the position on the VBGF curve at 24.3 m TL, which is the maximum size inferred for †*O. megalodon* in this study).

Shimada et al. (2021b) assessed the life history traits of †*Otodus megalodon* based on a growth parameter analysis using IRSNB P 9893 (Figure 1B, H–J) and assuming that it came from a 9.2-m-TL individual. In this study, we reassessed the ontogenetic parameters based on the new estimated TL of 16.4 m for IRSNB P 9893, where the outermost growth band (BN 46) was assumed to have formed when the shark was 16.4 m TL and died (Figure 6A). The estimated TL at the time of each grown band formation was then back-calculated based on the percentage distance from the center of the vertebral centrum (eTL in Table 4, Figure 6A). Forty-seven pairs of BN-TL values (including TL at BN 0) were obtained, and Figure 6B shows the VBGF fitted to correlate BN values with TL values. The VBGF parameters of the non-linear regression line (*r*^2^ = 99.9%; *p* < 0.001) are *L*_0_ = 385.794 cm TL, *L*_∞_ = 5,920.160 cm TL, and *k* = 0.00556 yr^–1^, and the longevity of the shark is calculated to be about 526.681 years (note that at least some of these values should not be taken at face value: see below). If each BN formed annually, other noticeable observations are that the estimated growth length gains (eGL in Table 4) in the first seven years (BN 0 through BN 7) range from 34 to 41 cm/yr with an average of 37.4 cm/yr, whereas the estimated growth length gains in the remaining 39 years (BN 8 through BN 46) range from 20 to 31 cm/yr with an average of 26.5 cm/yr.

The re-interpretation that IRSNB P 9893 measured about 16.4 m TL in life in this present study concomitantly alters some aspects of the proposed growth model of †*Otodus megalodon* significantly. Most notably, Shimada et al.’s (2021b) work suggested that the size at birth for †*O. megalodon* (or at least the individual represented by IRSNB P 9893) was about 2 m TL, but our back-calculation suggests that its size at birth was about 3.6 m TL (356 cm at BN 0 in Table 2) and possibly as much as about 3.9 m TL based on our new VBGF analysis (specifically *L*_0_ = 385.794 cm TL). The inferred large size at birth suggests that, like modern lamniforms (e.g., Gilmore et al., 2005), †*O. megalodon* practiced ovoviviparity with oophagous intrauterine cannibalism for embryonic nourishment (possibly besides lipid-rich uterine secretions or lipid histotrophy: see Sato et al., 2016; Shimada et al., 2021a), and neonates born with a competitive advantage and reduced predation risk, where one of the consequences of ovoviviparity with embryos’ oophagous behavior is low fecundity (generally one or two fetuses per each of the paired uteri: Shimada et al., 2021b). Although the exact size at sexual maturity can only be speculated for †*O. megalodon* at present, the fact that neonates of extant lamniforms are about 20−45% of the size of their mother depending on the species (Shimada et al., 2021a) suggests that the sexual maturity of female †*O. megalodon* may have been somewhere between 8 and 19.5 m TL to produce neonates measuring 3.6–3.9 m TL. Regardless, although neonates of whales can also be quite large with those of the extant blue whale *(Balaenoptera musculus)* measuring 7 m TL (Mizroch et al., 1984a), †*O. megalodon* may well represent the largest neonatal size at birth in the evolutionary history of non-tetrapod fishes, if its estimated size at birth of 3.6–3.9 m TL is indeed true.

Like this present study, Shimada et al. (2021b) suggested that the then-inferred size at birth of 2 m TL was large enough for neonates to have a highly competitive advantage and a low predation risk. This contention is even more true given our revised 3.6–3.9 m TL estimate for neonatal †*Otodus megalodon*. Curiously, the 3.6–3.9-m-TL neonatal size range corresponds to the size range of when extant *Carcharodon carcharias* undergoes a dietary shift from a predominantly fish diet to a largely marine mammal diet (see Tricas and McCosker, 1984; Klimley, 1985, 1994; McCosker, 1985; Long and Jones, 1996; Long et al., 1996; Estrada et al., 2006; Hussey et al., 2012). Therefore, it is conceivable that †*O. megalodon* was capable of taking large prey such as marine mammals upon birth. Furthermore, such a large neonatal size range, which would have already provided a competitive advantage and reduced predation risk relative to most other contemporaneous animals, seems to refute the hypothesis that †*O. megalodon* neonates utilized nursery areas (Purdy, 1996; Pimiento et al., 2010; Herraiz et al., 2020; for further discussion, see also Shimada et al., 2022).

The growth parameters based on a VBGF correlating BN values with TL values (Figure 6B) must be viewed as highly tentative or even improbable at least in part, not only because it is based on a single specimen but also because the VBGF curve beyond BN 46 is purely theoretical. This is particularly true considering that, where taxon-specific exceptions do exist, there is evidence to suggest that growth bands may simply record growth or vertebral size—i.e., not necessarily age or time—that may have led to systemic age underestimation in past growth studies of elasmobranchs (Passerotti et al., 2014; Harry, 2018; Natanson et al., 2018; Natanson and Deacy, 2019). The questionable nature of our VBGF parameters includes our *L*_∞_ value (5,920.160 cm), implying that †*Otodus megalodon* could have reached up to nearly 60 m TL with a longevity of about 527 years. Although the BN of 46 in this study may be underestimated and may not reflect the true age of IRSNB P 9893 at death, and although the oldest extant shark (Greenland shark: *Somniosus microcephalus*) ever recorded is estimated to be nearly 400 years old (Nielsen et al., 2016), our calculated longevity of over 500 years is most certainly an overestimation given that the maximum TL calibrated in this present study does not even exceed 25 m (see above). However, at least our estimated largest TL of 24.3 m TL would place the †*O. megalodon* individual to be about 83 years old (Figure 6B).

Like in Shimada et al.’s (2021b) study, our *k* value (0.00556 yr^–1^) is exceptionally low. The value must be interpreted with caution as with other VBGF parameters (see above), but if taken at face value, it suggests that it took a very long time for †*Otodus megalodon* to attain the mean maximum theoretical length. Shimada et al. (2021b) used lamnid sharks (e.g., *Isurus oxyrinchus* and *Lamna nasus*) that have the lowest *k* values (as low as 0.05−0.06) among extant lamniforms (Shimada, 2008) as a point of reference, which are still tenfold greater than the *k* value obtained for †*O. megalodon* even in the present study. Even if †*O. megalodon* never achieved the maximum theoretical length (e.g., *L*_∞_ = 5,920.160 cm), our nearly linear VBGF curve (Figure 6B) suggests that †*O. megalodon* showed relatively steady, indeterminate growth throughout its life, particularly after when BN 7 formed, with an average of 26.5 cm/yr (see above).

### Ecological and Evolutionary Implications

It must be emphasized that the findings and interpretations of the paleobiology of †*Otodus megalodon* presented here must be regarded as working hypotheses. This is 1) because they primarily rest on a single partial skeletal specimen from the Miocene of Belgium (IRSNB P 9893); and 2) because the general body size and morphology of †*O. megalodon* may have varied through time and space, where larger individuals appeared to have been more common in cooler waters compared to warmer waters (Shimada et al., 2022 vs. Pimiento and Balk, 2015). Additionally, the analytical approaches used in this study required us to make several assumptions about ontogenetic and evolutionary scaling of body and vertebral proportions with TL, which are at present untested. Furthermore, the present fossil record of †*O. megalodon* reveals practically nothing about its intraspecific, ontogenetic, and sexual variations of body properties, except the wide range of dental size and morphology. Nevertheless, it represents the only specimen suggesting that the species minimally attained 11.1 m, which does not even account for its head with a most likely massive jaw apparatus (e.g., Goto, 1989), nor for much of the caudal fin (Sternes et al., 2024). The fact that IRSNB P 9893 physically measures 11.1 m in length (Cooper et al., 2022), compared to the extrapolation based on the extant *Carcharodon carcharias* (Gottfried et al., 1996), led Sternes et al. (2024) to conclude that †*O. megalodon* must have had an elongated body relative to *C. carcharias*. This interpretation, in turn, implies that its pleuroperitoneal cavity was also elongated (Sternes et al., 2024), and as large ingested food items passed through its elongated digestive tract, its endothermic metabolism (Ferrón, 2017; Griffiths et al., 2023) likely facilitated their digestion, absorption, and further nutrient processing (Shimada et al., 2024a).

Along with its chronospecific predecessors (e.g., †*Otodus chubutensis*: Perez et al., 2019), †*O. megalodon* occupied a trophic position similar to (McCormack et al., 2022), or possibly even higher than (Kast et al., 2022), extant *Carcharodon carcharias* based on geochemical evidence, where the trace fossil record (e.g., tooth marks; sensu Zonneveld et al., 2022) suggests that its diet included marine mammals such as pinnipeds and cetaceans (e.g., Aguilera et al., 2008; Collareta et al., 2017a; Godfrey et al., 2018, 2021; Godfrey and Beatty, 2022; see also Paredes-Aliaga and Herraiz, 2024). If each growth band (Figure 6A) formed annually, our results show that the growth rate during the first seven years was relatively consistent with an average estimated rate of 37.4 cm/yr, and subsequently the relatively constant growth decreased to an average of 26.5 cm/yr. Although the rather constant gradual increase of TL in the first seven years is difficult to characterize as a “growth spurt”, the timing of the slightly reduced growth rate (around BN 7–8) marks shortly after the †*O. megalodon* individual had reached 6 m TL (Table 4; Figure 6B). Conclusions based only on a single specimen should be taken carefully; however, if IRSNB P 9893 is assumed to typify the growth rate of †*O. megalodon*, this TL-wise timing is intriguing ecologically and evolutionarily because it coincides well with the general size limit of 7 m TL throughout the history of macrophagous lamniforms (Shimada et al., 2021a). Macrophagous lamniforms over 6 m TL are considered “gigantic” where the only other possible genera contemporaneous with †*O. megalodon* (mid-Miocene–Early Pliocene) that could have included one or more members attaining such gigantic sizes are limited to *Alopias, Carcharodon, Isurus*, and †*Parotodus* (Kent, 1999; Shimada et al., 2021a, table 3, figure 4). Whereas †*O. megalodon*, which attained well beyond 6 m TL, is regarded as an extreme outlier not only for macrophagous lamniforms (Shimada et al., 2021a) but also for elasmobranchs in general (Pimiento et al., 2019), the fact that the aforementioned stock includes *Carcharodon* is of particular interest given that the rise of *C. carcharias* at the dawn of the Pliocene has been proposed to be at least in part responsible for the demise of †*O. megalodon* through competition (Boessenecker et al., 2019; McCormack et al., 2022). Based on IRSNB P 9893, the observed growth pattern of †*O. megalodon* appears as if it developed faster during the first seven years or so to “outgrow” the typical maximum size range of *Carcharodon* (e.g., while Pliocene *C. carcharias* possibly attained slightly larger sizes, the maximum size of extant *C. carcharias* is about 6.1 m TL: Randall, 1987; Castro, 2012; Collareta et al., 2023b) and then the growth rate decreased.

The timing of the slightly reduced growth rate at about 6.2–6.5 m TL in †*Otodus megalodon* (i.e., at BN 7 and 8 in Table 4) is even more intriguing from the standpoint of the evolution within the genus *Carcharodon*. During the Middle Miocene through the earliest Pliocene, *Carcharodon* was represented by †*C. hastalis*, which did not possess serrations on its tooth crowns, where a similar maximum TL to extant *C. carcharias* was likely already attained by †*C. hastalis* based on their comparable tooth sizes (e.g., de Muizon and DeVries, 1985; Cappetta, 2012). However, serrations began to develop in *Carcharodon* via †*C. hubbelli* in at least the Pacific Ocean around the Miocene–Pliocene transition (e.g., de Muizon and DeVries, 1985; Goto et al., 1993; Ehret et al., 2009a, 2009b, 2012, 2024). In addition, *C. carcharias* evolved a uniquely derived, mesial inclination of the cusp on the teeth of the upper third tooth row (i.e., the so-called “intermediate tooth row”: e.g., see Shimada, 2002, but also Siverson, 1999). The serrated teeth and upper intermediate teeth in *C. carcharias* have been regarded as derived traits promoting its feeding on large marine mammals (e.g., Martin et al., 2005; Ehret et al., 2012). Whereas piscivory in †*C. hastalis* has been demonstrated by a partial skeleton of a ca. 2.4-m-TL juvenile with bony fish remains as stomach contents (Collareta et al., 2017b), multiple cases of fossil cetacean bones with tooth marks putatively made by †*C. hastalis* are also known (Bianucci et al., 2010, 2018; Takakuwa, 2014; Bosio et al., 2021; Godfrey and Lowry, 2021). This indicates that the competition between the *Carcharodon* clade and †*O. megalodon* was likely already present in the Middle–Late Miocene marine ecosystems. Nitrogen and zinc isotopes analyzed in *Carcharodon* and †*Otodus* teeth offer additional possible support for the shifting trophic dynamics between these two lineages from the Miocene to the Pliocene–Recent (Kast et al., 2022; McCormack et al., 2022). Thus, the slightly faster growth rate during the first seven or eight years observed in IRSNB P 9893 (Table 4; Figure 6) could be interpreted as †*O. megalodon* rapidly “outgrowing” the typical maximum size range of †*C. hastalis* to be able to better compete for feeding on marine mammals. The level of interspecific competition between the two clades could have increased as serrations evolved and the shape and inclination of the crown in intermediate teeth changed markedly in the *Carcharodon* clade (i.e., in †*C. hubbelli*–*C. carcharias*), potentially allowing it to feed upon marine mammals more effectively than its predecessor. Whereas extant *C. carcharias* are known to feed on marine mammals, particularly larger individuals (e.g., Hussey et al., 2012, and references therein), multiple examples of Pliocene marine mammal bones showing serrated tooth marks and embedded serrated teeth of *Carcharodon* spp. have also been documented (Cigala-Fulgosi, 1990; Ehret et al., 2009b; Freschi and Cau, 2024; Godfrey et al., 2024, and references therein). In addition, although the study results are rather tenuous, a dental microwear analysis does not contradict the idea of competition between †*O. megalodon* and *C. carcharias* for marine mammals (Paredes-Aliaga and Herraiz, 2024).

Body temperature of †*O. megalodon* was comparable to, or possibly even slightly higher than, that of contemporaneous and extant *Carcharodon* spp. due to their regional endothermy (Griffiths et al., 2023, and references therein), but †*O. megalodon* is interpreted to have been no faster than *C. carcharias* in terms of cruising speed (see above). While climatic cooling and restructured ocean circulation during the Pliocene may also have had a role in †*O. megalodon*’s eventual demise (Condamine et al., 2019), the rise of smaller (thus likely more maneuverable) *C. carcharias* could have increased competition for prey consumed also by the presumably less agile †*O. megalodon*. Because the geographic range of †*O. megalodon* may have been decreasing across the Miocene–Pliocene transition (Pimiento et al., 2016), it is possible that †*O. megalodon* could have been already in decline before the first dispersal of *C. carcharias*. It should also be noted that the dispersal of *C. carcharias* from its Pacific stock was likely diachronous worldwide (see Boessenecker et al., 2019; Collareta et al., 2023b), suggesting that competitive exclusion may have been at play at different times in different regions of the global ocean. Furthermore, possible interactions of †*O. megalodon* with other contemporaneous large marine vertebrates, such as odontocetes as well as other sharks (including †*Parotodus benedenii*, another otodontid lamniform that likely reached at least 5 m TL [Shimada et al., 2021a, table 3] and possibly as much as 7.6 m TL [Kent, 1999; Collareta et al., 2023a]), should be explored. Nevertheless, besides the fact that the fossil record of †*O. megalodon* ends shortly after the emergence of *C. carcharias* (Boessenecker et al., 2019), it is noteworthy that this competitive exclusion scenario does have geochemical support (McCormack et al., 2022).

## Conclusions

This study indicates that the individual of †*Otodus megalodon* from the Miocene of Belgium represented by IRSNB P 9893 (with a maximum vertebral diameter of 15.5 cm) possibly measured about 16.4 m TL and weighed around 30 t. Although highly inferential, this study also shows that †*O. megalodon* could have attained at least about 24.3 m TL and weighed about 94 t based on the gigantic vertebral specimen from the Miocene of Denmark reported by Bendix-Almgreen (1983). It must be emphasized that these TL estimates are based on a new method that is completely independent of the traditional tooth-based approach and does not assume *Carcharodon carcharias* as a modern analog like the previous studies (e.g., Randall, 1973; Gottfried et al., 1996; Shimada, 2003, 2019; Reolid and Molina, 2015; Razak and Kocsis, 2018; Cooper et al., 2020, 2022; Perez et al., 2021).

Our cluster analysis conducted on three body part proportions (NP, TP, and CP: Figure 2) suggests that the body form of †*Otodus megalodon* could have indeed been slenderer than the previous reconstructions of the fossil shark (e.g., Gottfried et al., 1996; Cooper et al., 2020, 2022). Our allometric analysis (Figure 4) further shows that sharks with positive allometric growth, such as *Lamna nasus* and *Carcharodon carcharias*, likely cannot achieve gigantism, whereas sharks that achieve large or gigantic sizes *(Cetorhinus, Megachasma, Rhincodon*, and *Somniosus)* have body forms that conform to those of most large cetaceans with high fineness ratios. Although body forms of previous †*O. megalodon* reconstructions are still theoretically possible, our study suggests that they would have been hydrodynamically challenged and could not have resulted in an efficient swimmer at the body size values typical of adult †*O. megalodon*. On the other hand, the trend seen across *Cetorhinus, Megachasma, Rhincodon*, and *Somniosus* (Figure 4C) indicates that †*O. megalodon* could have grown gigantic (e.g., 24.3 m TL) while still maintaining hydrodynamic efficiency as in gigantic cetaceans such as *Balaenoptera* spp. Our study suggests that giant †*O. megalodon* possibly had a fineness ratio of about 6 (Figure 5C), supporting the general conclusion made by Sternes et al. (2024) that †*O. megalodon* must have had a slenderer body compared to *C. carcharias* and other lamnids. As a matter of fact, there is currently no evidence whatsoever that †*O. megalodon* resembled *C. carcharias* on which the previous reconstructions were mostly based (e.g., Gottfried et al., 1996; Cooper et al., 2020, 2022).

Our study based on IRSNB P 9893 suggests that the size at birth of †*Otodus megalodon* was about 3.6–3.9 m TL, quite possibly marking the largest neonate size in the evolutionary history of fishes. The large neonate size strongly supports the interpretation that †*O. megalodon* was ovoviviparous where embryos likely exhibited oophagous intrauterine cannibalism for nourishment before their birth. Although purely inferential based on extant lamniforms, the estimated size at sexual maturity of female †*O. megalodon* was possibly somewhere between 8 and 19.5 m TL. If taken at face value, our VBGF analysis based on the growth bands observed in IRSNB P 9893 implies that the ontogenetic growth of †*O. megalodon* was overall slow and relatively steady to attain the mean maximum theoretical length, where our estimated maximum TL of 24.3 m TL would place the shark to be about 83 years old. However, a slight decrease in growth rate was noted at about age 7 from 37.4 cm/yr to 26.5 cm/yr on average. Where its large size at birth offers not only a low predation risk, which seems to further refute the idea that neonate †*O. megalodon* utilized nursery areas (Shimada et al., 2022; vs. Pimiento et al., 2010; Herraiz et al., 2020), but also a high competitive advantage already capable of feeding on marine mammals, this decrease in growth rate marks the time when the shark had reached slightly over 6 m TL (Table 4; Figure 6B). This size coincides well with the general size limit of 7 m TL in macrophagous lamniforms (Shimada et al., 2021a), including the precursor of *Carcharodon carcharias* via †*C. hubbelli*, †*C. hastalis*, which lived contemporaneously alongside †*O. megalodon* during the Middle–Late Miocene. Whereas the teeth of †*C. hastalis* are unserrated, †*C. hubbelli* and *C. carcharias* acquired serrations and eventually also modified the shape of the upper third tooth (“intermediate tooth row”), resulting in a uniquely-derived mesially directed cusp—traits that appear to be associated with feeding upon large marine mammals (e.g., Martin et al., 2005; Ehret et al., 2012). Therefore, the observed growth pattern of †*O. megalodon* based on IRSNB P 9893 is that it seems to have developed faster during the first seven years or so to “outgrow” the typical maximum size range of *Carcharodon* to reduce competition during the Middle–Late Miocene. However, supporting an idea previously proposed based on biostratigraphic (Boessenecker et al., 2019) and geochemical (McCormack et al., 2022) evidence, the evolution of serrated *Carcharodon* around the Miocene-Pliocene transition could have led to increased competition for marine mammals between †*O. megalodon* and *Carcharodon*, where agile *C. carcharias* could have contributed to the decline of the less maneuverable †*O. megalodon* through competitive exclusion.

The paleobiological interpretations of †*Otodus megalodon* presented in this study must be viewed as working hypotheses. The fact is that the present fossil record still does not reveal exactly how large †*O. megalodon* was beyond the 11.1 m partial vertebral column (IRSNB P 9893; Figure 1B) or what exactly †*O. megalodon* looked like. It should be added that, in reality, even the taxonomic identity of IRSNB P 9893 as †*O. megalodon* remains inferential because it was not associated with any teeth (see Gottfied et al., 1996). Arguably, the next best specimen of †*O. megalodon* is that from the Miocene of Japan consisting of associated teeth, fragments of tessellated calcified cartilage, and placoid scales (Uyeno et al., 1989; Shimada et al. 2024a, 2024b); however, it does not come with any vertebrae. Therefore, the discovery of more and better-preserved skeletal remains of †*O. megalodon*, preferably with associated teeth and other anatomical elements, is needed to empirically address issues concerning its body size and body form.

In the context of estimating the maximum body size of †*Tyrannosaurus rex*, Mallon and Hone (2024, p. 8) stated that “There is, inevitably, great popular and scientific interest in the extremes of large body size” (see also Gayford et al., 2024b). This present study is no exception where we presented the estimated maximum possible TL of †*Otodus megalodon* by noting the importance of deciphering the body size of large extinct animals in the context of ecology and evolution. However, the primary purpose of this study was to reassess the basic biology of †*O. megalodon* in hopes of clarifying some existing misunderstandings or misconceptions about the fossil shark. Similar to Mallon and Hone’s (2024, p. 8) sentiment, we note that the rigid pursuit to determine popular questions among the general public as to exactly how large †*O. megalodon* could have grown or how exactly it compared to (or if it was “stronger” than), for example, †*Livyatan melvillei* (a gigantic contemporaneous toothed whale) is not necessarily productive, particularly given the limited number of meaningful †*O. megalodon* fossils. Such a quest can obscure more interesting or important scientific questions stemming from the great diversity and evolutionary history of sharks and all other coexisting organisms. This is particularly true because, if interpreted correctly, understanding the mechanisms and consequences of evolution and extinction of prehistoric organisms, including †*O. megalodon*, will allow us to make predictions about how present-day organisms may respond to major shifts in climate, environment, and biodiversity that are critical for conservation biology of living species, including ecologically critical sharks (e.g., Pimiento and Antonelli, 2022, and references therein; Dedman et al., 2024; Dulvy et al., 2024).

## Supplementary Material

Appendix

## Figures and Tables

**Table 1:** List of all extant and extinct lamniform species examined in this study with their family, maximum total length for extant taxa (mTL; in cm; based on Ebert et al., 2021), neurocranial proportion (NP), trunk proportion (TP), and caudal fin proportion (CP) as well as “adjusted neurocranial proportion” (aNP) and “adjusted caudal fin proportion” (aCP) (see Figure 1C and text). This table also gives a median value for each variable.

Species	Family	mTL	NP	TP	CP	aNP	aCP
†*Palaeocarcharias stromeri*	†Palaeocarchariidae*	-	0.108	0.670	0.222	0.161	0.331
†*Scapanorhynchus lewisii*	Mitsukurinidae	-	0.175	0.507	0.318	0.345	0.627
*Mitsukurina owstoni*	Mitsukurinidae	620	0.205	0.478	0.317	0.429	0.663
†*Aquilolamna milarcae*	†Aquilolamnidae	-	0.067	0.624	0.309	0.107	0.495
†*Ptychodus* sp.	†Ptychodontidae	-	0.126	0.697	0.177	0.181	0.254
*Carcharias taurus*	Carchariidae	325	0.105	0.631	0.264	0.166	0.418
*Odontaspis ferox*	Odontaspididae	450	0.131	0.607	0.262	0.216	0.432
*Odontaspis noronhai*	Odontaspididae	427	0.122	0.606	0.272	0.201	0.449
*Pseudocarcharias kamoharai*	Pseudocarchariidae	122	0.126	0.662	0.212	0.190	0.320
*Megachasma pelagios*	Megachasmidae	820	0.097	0.588	0.315	0.165	0.536
*Cetorhinus maximus*	Cetorhinidae	1097	0.092	0.714	0.194	0.129	0.272
*Alopias pelagicus*	Alopiidae	428	0.059	0.400	0.541	0.148	1.353
*Alopias superciliosus*	Alopiidae	484	0.071	0.501	0.428	0.142	0.854
*Alopias vulpinus*	Alopiidae	575	0.056	0.416	0.525	0.135	1.262
*Carcharodon carcharias*	Lamnidae	640	0.119	0.690	0.191	0.166	0.275
*Isurus oxyrinchus*	Lamnidae	445	0.129	0.695	0.176	0.186	0.253
*Isurus paucus*	Lamnidae	430	0.108	0.681	0.211	0.159	0.310
*Lamna ditropis*	Lamnidae	305	0.107	0.695	0.198	0.154	0.285
*Lamna nasus*	Lamnidae	365	0.111	0.668	0.221	0.166	0.331
MEDIAN VALUES			0.108	0.631	0.262	0.166	0.418

* Ordinal and familial assignments of this species are questionable (see Villalobos-Segura et al., 2023)

**Table 2:** List of all extant and extinct orders as well as an operational category examined in this study and their minimum (Min.) and maximum (Max.) “adjusted neurocranial proportion” (aNP) and “adjusted caudal fin proportion” (aCP) as well as median aNP and aCP (see Figure 1C and text; data based on Appendix 3).

Taxon or examined category	Min. aNP	Max. aNP	Median aNP	Min. aCP	Max. aCP	Median aCP
†Synechodontiformes	na	na	0.268^b^	na	na	0.720^b^
Hexanchiformes	0.058	0.233	0.173	0.391	0.559	0.464
Echinorhiniformes	na	na	0.214^b^	na	na	0.361^b^
Squaliformes	0.086	0.336	0.189	0.190	0.493	0.302
Pristiophoriformes	0.401	0.657	0.533	0.265	0.339	0.299
Squatiniformes	0.122	0.131	0.126	0.163	0.176	0.170
†*Protospinax annectans*^a^	na	na	0.234^c^	na	na	0.226^c^
Heterodontiformes	0.180	0.211	0.188	0.262	0.333	0.295
Orectolobiformes	0.106	0.305	0.166	0.186	1.130	0.327
Lamniformes	0.107	0.429	0.166	0.253	1.353	0.418
Lamniformes minus mitsukurinids and alopiids	0.107	0.216	0.166	0.253	0.536	0.326
Carcharhiniformes	0.107	0.396	0.189	0.203	0.615	0.354
ALL EXAMINED SPECIES COMBINED	0.058	0.657	0.183	0.163	1.353	0.333

a Order incertae sedis;

b n=1;

c average of n = 3

**Table 3:** Reported cruising speeds (CS; in km h^-1^; mean value is given if n>1) of select sharks and whales discussed in the text.

Group	Species (common name)	n	CS	Source
Sharks				
	*Cetorhinus maximus* (basking shark)	21*	3.9	Sims (2000)
	*Megachasma pelagios* (megamouth shark)	1	1.5	Nelson et al. (1997)
	*Rhincodon typus* (whale shark)	12	3.1	Gleiss et al. (2011)
	*Somniosus microcephalus* (Greenland shark)	6	1.3	Watanabe et al. (2012)
Whales				
	*Balaenoptera borealis* (sei whale)	1	8.0	Gough et al. (2021)
	*Balaenoptera musculus* (blue whale)	17	7.9	Gough et al. (2021)
	*Balaenoptera physalus* (fin whale)	2	10.4	Gough et al. (2021)
	*Eubalaena glacialis* (North Atlantic right whale)	29	1.9**	Hain et al. (2013)
	*Megaptera novaeangliae* (humpback whale)	29	7.5	Gough et al. (2021)
	*Physeter macrocephalus* (sperm whale)	137	6.4	Aoki et al. (2007)

* A total of 21 ‘speed determinations’ from six individuals.

** Data of ‘singles and non-mother calf’ that consisted of a group with the fastest CS in their dataset.

**Table 4:** Raw measurements (BN, CR, and BI: from Shimada et al., 2021b, table 1) and derived measurements (pCR, eTL, and eGL) based on the sectioned vertebra of †*Otodus megalodon* (IRSNB P 9893, “centrum #4”; Figure 1J), where all data come from Shimada et al. (2021b, table 1) except eTL and eGL that are based on the new interpretation that IRSNB P 9893 measured 16.4 m TL when it died. Abbreviations: BN, band number; CR, centrum radius; BI, band interval from the previous band; pCR, percent centrum radius from the center of the vertebra; eTL, extrapolated total length of entire shark; eGL, estimated GL, estimated growth length gain from the previous year.

BN	CR (mm)	BI (mm)	pCR (%)	eTL (cm)	eGL (cm)
0	16.8	-	21.7	356	-
1	18.7	1.9	24.1	395	39
2	20.5	1.8	26.4	433	38
3	22.4	1.9	28.9	474	41
4	24.2	1.8	31.2	512	38
5	25.8	1.7	33.3	546	34
6	27.6	1.8	35.6	584	38
7	29.2	1.6	37.7	618	34
8	30.7	1.5	39.6	649	31
9	32.0	1.3	41.3	677	28
10	33.3	1.3	43.0	705	28
11	34.6	1.3	44.6	731	26
12	35.7	1.1	46.0	754	23
13	36.8	1.1	47.4	777	23
14	37.9	1.1	48.9	802	25
15	39.1	1.2	50.4	827	25
16	40.0	1.0	51.7	848	21
17	41.2	1.2	53.2	872	25
18	42.6	1.4	55.0	902	30
19	43.9	1.3	56.7	930	28
20	45.2	1.3	58.3	956	26
21	46.5	1.3	60.0	984	28
22	47.8	1.2	61.7	1012	28
23	49.1	1.3	63.3	1038	26
24	50.0	1.4	65.2	1069	31
25	51.9	1.4	67.0	1099	30
26	53.4	1.5	68.9	1130	31
27	54.8	1.4	70.7	1159	30
28	56.1	1.3	72.4	1187	28
29	57.5	1.4	74.2	1217	30
30	58.9	1.4	76.0	1246	30
31	59.9	1.0	77.3	1268	21
32	61.0	1.1	78.7	1291	23
33	62.0	1.0	80.0	1312	26
34	63.1	1.0	81.4	1335	30
35	64.3	1.2	83.0	1361	23
36	65.7	1.4	84.8	1391	30
37	66.8	1.0	86.2	1414	23
38	67.8	1.0	87.4	1433	20
39	69.1	1.3	89.2	1463	30
40	70.4	1.3	90.8	1489	26
41	71.6	1.2	92.4	1515	26
42	72.8	1.2	94.0	1542	26
43	74.0	1.2	95.5	1566	25
44	75.1	1.1	96.9	1589	23
45	76.3	1.1	98.4	1614	25
46	77.5	1.2	100.0	1640	26
