# Supplementary material for: Reassessment of the possible size, form, weight, cruising speed, and growth parameters of the extinct megatooth shark, Otodus megalodon (Lamniformes: Otodontidae), and new evolutionary insights into its gigantism, life history strategies, ecology, and extinction
Source: Palaeontol Electronica. Author manuscript; Available in PMC 2025 Mar 13. (PMC7617484; doi:10.26879/1502)
Supplement: Appendix [file EMS203651-supplement-Appendix.pdf]

APPENDICES

APPENDIX 1.

List of all extant shark species for which their neurocranial length (NL; in cm) was measured in this study along with their source, sex, total length (TL; in cm), and neurocranial proportion (NP = NL/TL) (see Figure 1C and text) (see Materials and Methods for list of repository institutions for listed voucher specimens). Asterisk (*) indicates the average value.

Species name	Source	Sex	TL	NL	NP
Chlamydoselachus anguineus	Shirai, 1992, pl. 1A, B	M	139	5.5	0.040
Heptranchias perlo	Goto, 2001, fig. 3A	M	84	8.4	0.100
Hexanchus griseus	Shirai, 1992, pl. 2A, B	M	70	7.8	0.111
Notorynchus cepedianus	SIO 82-62	F	89	11.7	0.130
Echinorhinus cookei	LACM 33827.031	M	66	9.0	0.136
Cirrhigaleus barbifer	Shirai, 1992, pl. 12A-B	F	58	7.0	0.121
Squalus mitsukurii	Viana and Soares, 2023, fig. 2E	M	77	11.9	0.155
Centrophorus granulosus	Shirai, 1992, pl. 11D	M	66	11.1	0.168
Deania calcea	Shirai, 1992, pl. 11A, B	F	59	11.1	0.188
Aculeola nigra	SIO 72-167	M	54	3.7	0.070
Centroscyllium excelsum	Shirai, 1992, pl. 4A, B	M	59	8.8	0.149
Etmopterus lucifer	Staggl et al., 2023, fig. 3C, D	M	35	5.3	0.151
Trigonognathus kabeyai	Shirai, 1992, pl. 5A, B	F	26	2.7	0.104
Centroscymnus owstonii	SIO 72-156	M	73	8.6	0.118
Centroselachus crepidater	USNM 94522	?	130	7.9	0.061
Scymnodalatias sherwoodi	USNM RAD100101	?	80	4.9	0.061
Somniosus microcephalus	White, 1895, pl. 1, fig. 1, 2	?	198	16.9	0.085
Zameus squamulosus	Shirai, 1992, pl. 6A, B	M	53	7.3	0.138
Oxynotus bruniensis	Shirai, 1992, pl. 7A, B	M	55	6.8	0.124
Dalatias licha	Shirai, 1992, pl. 8A, B	M	39	4.9	0.126
Euprotomicrus bispinatus	USNM 190031	?	31	2.5	0.081
Heteroscymnoides marleyi	ANSP 53046	?	29	5.8	0.200
Isistius brasiliensis	Shirai, 1992, pl. 9A, B	F	52	6.0	0.115
Mollisquama sp.	Denton et al., 2018, fig. 1A, B	M	14	1.0	0.071
Squaliolus laticaudus	Shirai, 1992, pl. 10A, B	F	25	4.9	0.196
Pliotrema annae	Weigmann et al., 2020, fig. 12B	F	98	25	0.255
Pliotrema kajae	Weigmann et al., 2020, fig. 12A	F	56	17	0.304
Pliotrema warreni	Weigmann et al., 2020, fig. 31	F	70	23	0.329
Pristiophorus japonicus	SIO 92-164	F	113	32.6	0.288
Pristiophorus nudipinnis	Mollen et al., 2016, fig. 3B, C	F	83	19.6	0.236
Squatina africana	Mollen et al., 2016, fig. 4A, E	M	82	7.8	0.095
Squatina japonica	Shirai, 1992, pl. 13A, B	M	51	5.1	0.100
Heterodontus francisci	SIO 64-33	F	77	9.2	0.119
Heterodontus zebra	Shirai, 1992, pl. 16A, B	M	44	6.3	0.143
Cirrhoscyllium japonicum	Goto, 2001, 12B, C	F	44	3.8	0.086
Parascyllium ferrugineum	Goto, 2001, fig. 11B, C	M	70	5.7	0.081
Brachaelurus waddi	Goto, 2001, fig, 8B, C	F	28	3.2	0.114

Species name	Source	Sex	TL	NL	NP
Eucrossorhinus dasypogon	BMNH 1867	M	15	2.8	0.187
Orectolobus ornatus	Goto, 2001, 9B, C	F	26	3.0	0.115
Orectolobus wardi	Goto, 2001, fig. 10B, C	F	34	3.9	0.115
Sutorectus tentaculatus	YPM 006167	M	24	3.1	0.129
Chiloscyllium plagiosum	Goto, 2001, fig. 6B, C	F	74	8.0	0.108
Hemiscyllium freycineti	Goto, 2001, fig. 7B, C	M	19	1.8	0.095
Stegostoma tigrinum	UMMZ 219891	F	46	5.4	0.117
Pseudoginglymostoma brevicaudatum	ZMUC 2396735	F	53	4.9	0.092
Ginglymostoma cirratum	Goto, 2001, fig. 14B, C	F	35	4.3	0.123
Rhincodon typus	Goto, 2001, fig. 15B, C	M	481	38.5	0.080
Mitsukurina owstoni 1	FMNH 117742	F	127	26.5	0.209
Mitsukurina owstoni 2	SIO 07-46	M	115	23.0	0.200
*Mitsukurina owstoni**	-	-	-	-	0.205*
Carcharias taurus 1	FMNH 16136	M	106	11.0	0.104
Carcharias taurus 2	MCZ 436	F	100	10.5	0.105
*Carcharias taurus**	-	-	-	-	0.105*
Odontaspis ferox 1	BPBM 9334	F	190	25.0	0.132
Odontaspis ferox 2	BPBM 9335	?	297	38.5	0.130
*Odontaspis ferox**	-	-	-	-	0.131*
Odontaspis noronhai 1	HUMZ 110959	M	217	26.0	0.120
Odontaspis noronhai 2	TCWC 3922	F	321	39.5	0.123
*Odontaspis noronhai**	-	-	-	-	0.122*
Pseudocarcharias kamoharai 1	FMNH 117474	M	101	12.5	0.124
Pseudocarcharias kamoharai 2	LACM 45857	F	92	11.5	0.125
Pseudocarcharias kamoharai 3	USNM 303206	M	93	12.0	0.129
*Pseudocarcharias kamoharai**	-	-	-	-	0.126*
Megachasma pelagios 1	SIO 07-53	F	215	16.0	0.074
Megachasma pelagios 2	Compagno, 1990, figs. 5E, 6E	M	446	53.4	0.120
*Megachasma pelagios**	-	-	-	-	0.097*
Cetorhinus maximus 1	MCZ 54413	F	385	35.0	0.091
Cetorhinus maximus 2	Compagno, 1990, figs. 5F, 6F	M	701	65.1	0.093
*Cetorhinus maximus**	-	-	-	-	0.092*
Alopias pelagicus 1	FMNH 117473	F	169	10.0	0.059
Alopias superciliosus 1	UF 160188	M	187	13.0	0.070
Alopias superciliosus 2	UF 178509	M	201	14.5	0.072
*Alopias superciliosus**	-	-	-	-	0.071*
Alopias vulpinus 1	SIO 64-804	M	145	8.5	0.059
Alopias vulpinus 2	SIO 78-138	M	131	7.0	0.053
*Alopias vulpinus**	-	-	-	-	0.056*
Carcharodon carcharias 1	LACM 43805-1	M	126	16.5	0.131
Carcharodon carcharias 2	LACM 56960-1	F	209	21.5	0.103
Carcharodon carcharias 3	MCZ 164195	?	260	34.0	0.131
Carcharodon carcharias 4	Mollen et al., 2012, table 1, fig. 2B	F	212	20.3	0.096
*Carcharodon carcharias**	-	-	-	-	0.115*
Isurus oxyrinchus 1	UMMZ 179082	?	185	25.5	0.138

Species name	Source	Sex	TL	NL	NP
Isurus oxyrinchus 2	UMMZ 94726	M	85	11.0	0.129
Isurus oxyrinchus 3	Mollen et al., 2012, table 1, fig. 3B	F	194	23.4	0.121
*Isurus oxyrinchus**	-	-	-	-	0.129*
Isurus paucus 1	UF 160174	M	125	13.5	0.108
Isurus paucus 2	Mollen et al., 2012, table 1, fig. 4B	F	254	27.2	0.107
*Isurus paucus**	-	-	-	-	0.108*
Lamna ditropis 1	FMNH 117475	M	151	18.0	0.119
Lamna ditropis 2	Mollen et al., 2012, table 1, fig. 6B	F	234	18.9	0.081
Lamna ditropis 3	Mollen et al., 2012, table 1	F	90	10.8	0.120
*Lamna ditropis**	-	-	-	-	0.107*
Lamna nasus 1	MCZ 37028	M	115	14.0	0.122
Lamna nasus 2	Mollen et al., 2012, table 1, fig. 5B	M	174	18.9	0.109
Lamna nasus 3	Mollen et al., 2012, table 1	M	166	16.7	0.101
*Lamna nasus**	-	-	-	-	0.111*
Apristurus laurussonii	Compagno, 1988, fig. 13.36	M	30	6.3	0.210
Asymbolus vincenti	Compagno, 1988, fig. 13.16	M	50	5.0	0.100
Bythaelurus canescens	Compagno, 1988, fig. 13.25	F	60	7.6	0.127
Cephalurus sp.	Compagno, 1988, fig. 13.18	F	30	3.6	0.120
Figaro boardmani	CSIRO H 3684-02	M	53	6.8	0.128
Galeus arae	Compagno, 1988, fig. 13.21	F	32	4.3	0.134
Halaelurus buergeri	Compagno, 1988, fig. 13.24	M	36	4.1	0.114
Haploblepharus fuscus	Compagno, 1988, fig. 13.27	F	61	5.9	0.097
Holohalaelurus regani	Compagno, 1988, fig. 13.29	M	56	5.7	0.102
Parmaturus pilosus	Compagno, 1988, fig. 13.31	F	47	4.7	0.100
Parmaturus xaniurus	Compagno, 1988, fig. 13.32	M	43	5.4	0.126
Pentanchus profundicolus	USNM 70260	?	51	7.2	0.141
Dichichthys bigus	White et al., 2024, figs. 2c, 9	F	71	9.7	0.137
Atelomycterus marmoratus	Compagno, 1988, fig. 13.2	F	44	4.3	0.098
Aulohalaelurus labiosus	Compagno, 1988, fig. 13.4	M	62	6.0	0.097
Cephaloscyllium ventriosum	Compagno, 1988, fig. 13.10	F	76	9.1	0.120
Poroderma africanum	Compagno, 1988, fig. 13.12	M	87	9.2	0.106
Schroederichthys chilensis	Compagno, 1988, fig. 13.6	F	34	3.8	0.112
Schroederichthys maculatus	Compagno, 1988, fig. 13.7	M	31	3.3	0.106
Scyliorhinus retifer	Compagno, 1988, fig. 13.13	F	30	4.3	0.143
Ctenacis fehlmanni	Compagno, 1988, fig. 14.1	F	46	5.7	0.124
Eridacnis barbouri	Compagno, 1988, fig. 14.3	F	28	3.8	0.136
Proscyllium habereri	Shirai, 1992, pl. 17A, B	F	54	5.5	0.102
Gollum attenuatus	Compagno, 1988, fig. 14.5	M	96	15.3	0.159
Planonasus parini	ZMH 25895	F	52	5.1	0.098
Pseudotriakis microdon	Compagno, 1988, fig. 15.3	M	197	21.4	0.109
Leptocharias smithii	Compagno, 1988, fig. 16.1	F	52	6.0	0.115
Furgaleus macki	Compagno, 1988, fig. 17.14	F	19	2.9	0.153
Gogolia filewoodi	Compagno, 1988, fig. 6.2A, B	?	22	4.1	0.186
Galeorhinus galeus	Compagno, 1988, fig. 17.18	M	73	12.4	0.170
Hemitriakis japanica	Compagno, 1988, fig. 17.12	M	65	8.2	0.126

Species name	Source	Sex	TL	NL	NP
Hypogaleus hypugaensis	Compagno, 1988, fig. 17.17	F	115	11.3	0.098
Iago omanesis	Compagno, 1988, fig. 17.15	F	58	6.7	0.116
Mustelus californicus	Compagno, 1988, fig. 17.7B	F	121	9.5	0.091
Mustelus fasciatus	Compagno, 1988, fig. 17.7E	M	58	8.7	0.150
Mustelus higmani	Compagno, 1988, fig. 17.7D	M	45	5.8	0.129
Mustelus lenticulatus	Compagno, 1988, fig. 17.7F	M	93	11.7	0.126
Mustelus lunulatus	Compagno, 1988, fig. 17.6	F	97	11.2	0.115
Mustelus mosis	Compagno, 1988, fig. 17.7C	F	82	10.0	0.122
Mustelus whitneyi	Compagno, 1988, fig. 17.7A	F	79	9.7	0.123
Scylliogaleus quecketti	Compagno, 1988, fig. 17.10	F	86	9.1	0.106
Triakis acutipinna	Compagno, 1988, fig. 17.4B	M	90	9.2	0.102
Triakis maculata	Compagno, 1988, fig. 17.4C	F	43	5.9	0.137
Triakis megalopterus	Compagno, 1988, fig. 17.4D	M	76	8.4	0.111
Triakis scyllium	Compagno, 1988, fig. 17.4A	M	109	10.0	0.092
Triakis semifasciata	Compagno, 1988, fig. 17.3	F	94	9.3	0.099
Chaenogaleus macrostoma	Compagno, 1988, fig. 18.6	M	65	6.8	0.105
Hemigaleus microstoma	Compagno, 1988, fig. 18.3	F	43	5.7	0.133
Hemipristis elongata	Compagno, 1988, fig. 18.7	F	170	16	0.094
Paragaleus tengi	Compagno, 1988, fig. 18.1	M	85	9.3	0.109
Carcharhinus amblyrhynchoides	Compagno, 1988, fig. 19.10A	M	140	17	0.121
Carcharhinus amboinensis	Compagno, 1988, fig. 19.10L	M	72	10.3	0.143
Carcharhinus borneensis	Compagno, 1988, fig. 19.10H	F	47	7.2	0.153
Carcharhinus falciformis	Compagno, 1988, fig. 19.10B	F	124	17.3	0.140
Carcharhinus galapagensis	Compagno, 1988, fig. 19.10D	F	100	11.7	0.117
Carcharhinus hemiodon	Compagno, 1988, fig. 19.10I	M	55	8.4	0.153
Carcharhinus isodon	Compagno, 1988, fig. 19.10E	M	52	7.8	0.150
Carcharhinus limbatus	Compagno, 1988, fig. 19.10N	?	120	14.4	0.120
Carcharhinus longimanus	Compagno, 1988, fig. 19.10P	F	124	15.7	0.127
Carcharhinus macloti	Compagno, 1988, fig. 19.9	F	52	8.5	0.163
Carcharhinus melanopterus	Compagno, 1988, fig. 19.10M	F	123	17.8	0.145
Carcharhinus obscurus	Compagno, 1988, fig. 19.8	?	140	14.8	0.106
Carcharhinus porosus	Compagno, 1988, fig. 19.10G	?	120	16.8	0.140
Carcharhinus sealei	Compagno, 1988, fig. 19.10F	M	74	8.8	0.119
Carcharhinus sorrah	Compagno, 1988, fig. 19.10J	M	110	13.2	0.120
Glyphis sp.	Compagno, 1988, fig. 19.13	M	72	9.6	0.133
Isogomphodon oxyrhynchus	Compagno, 1988, fig. 19.6	F	62	11.5	0.185
Lamiopsis temmincki	Compagno, 1988, fig. 19.14	F	40	5.6	0.140
Loxodon macrorhinus	Compagno, 1988, fig. 19.3	F	89	9.1	0.102
Negaprion brevirostris	Compagno, 1988, fig. 19.17	?	140	15.6	0.111
Nasolamia velox	Compagno, 1988, fig. 19.16	M	53	9.5	0.179
Prionace glauca	Compagno, 1988, fig. 19.18	M	151	19.5	0.129
Rhizoprionodon acutus	Compagno, 1988, fig. 19.5B	F	72	9.4	0.131
Rhizoprionodon lalandii	Compagno, 1988, fig. 19.5A	M	45	6.6	0.147
Rhizoprionodon longurio	Compagno, 1988, fig. 19.4	F	50	8.7	0.174
Rhizoprionodon oligolinx	Compagno, 1988, fig. 19.5C	F	57	7.4	0.130

Species name	Source	Sex	TL	NL	NP
Rhizoprionodon terraenovae	Compagno, 1988, fig. 19.5D	?	80	10.8	0.125
Scoliodon laticaudus	Compagno, 1988, fig. 19.2	F	66	10.5	0.159
Triaenodon obesus	Compagno, 1988, fig. 19.20	?	140	13.7	0.098
Galeocerdo cuvier	Compagno, 1988, fig. 19.1	F	138	13.9	0.101
Eusphyra blochii	Compagno, 1988, fig. 20.5	M	132	9.5	0.072
Sphyrna lewini	SIO 87-120	M	75	4.99	0.066
Sphyrna tiburo	Compagno, 1988, fig. 20.1	F	80	9.0	0.113

APPENDIX 2.

List of extinct neoselachian shark taxa examined in this study along with their age (J, Jurassic; C, Cretaceous), source, neurocranial proportion (NP), trunk proportion (TP), and caudal proportion (CP). Asterisk (*) indicates the average value.

Species name (Age)	Age	Source	NP	TP	CP
†*Paraorthacodus* sp.	J	Kriwet and Klug, 2004, fig. 7a	0.135	0.503	0.362
†*Centrosqualus primaevus*	C	Cappetta, 1980, pl. 5, fig. 1	0.118	0.684	0.198
†*Cretascymnus adonis*	C	Cappetta, 1980, pl. 4, fig. 1	0.140	0.723	0.137
†*Synechodus* sp.	J	Thies and Leidner, 2011, pl. 62, fig. A	0.126	0.604	0.270
†*Pseudorhina acanthoderma*	J	Thies and Leidner, 2011 pl. 15, fig. A	0.096	0.791	0.113
†*Pseudorhina alifera*	J	Kriwet and Klug, 2004, fig. 12a	0.091	0.735	0.174
†*Protospinax annectans* 1	J	Jambura et al., 2023, fig. 1A	0.157	0.685	0.158
†*Protospinax annectans* 2	J	Jambura et al., 2023, fig. 1C	0.164	0.684	0.152
†*Protospinax annectans**	J	-	0.160*	0.685*	0.155*
†*Heterodontus zitteli*	J	Thies and Leidner, 2011, pl. 28, fig. A	0.124	0.671	0.205
†*Paracestracion falcifer* 1	J	Kriwet and Klug, 2004, fig. 12a	0.144	0.667	0.189
†*Paracestracion falcifer* 2	J	Thies and Leidner, 2011, pl. 27, fig. A	0.114	0.691	0.195
†*Paracestracion falcifer**	J	Thies and Leidner, 2011, pl. 27, fig. A	0.129*	0.679*	0.192
†*Mesiteia emiliae* 1	C	Pfeil, 2021, pl.1, fig.4	0.082	0.774	0.144
†*Mesiteia emiliae* 2	C	Pfeil, 2021, pl.1, fig.5	0.083	0.774	0.143
†*Mesiteia emiliae**	C	-	0.082*	0.774*	0.144*
†*Phorcynis catulina*	J	Thies and Leidner, 2011, pl. 33, fig. A	0.118	0.664	0.218
†*Palaeocarcharias stromeri* 1	J	Kriwet and Klug, 2004, fig. 15b	0.100	0.691	0.209
†*Palaeocarcharias stromeri* 2	J	Duffin, 1988, fig. 1A	0.106	0.632	0.262
†*Palaeocarcharias stromeri* 3	J	Duffin, 1988, fig. 1B	0.117	0.688	0.195
†*Palaeocarcharias stromeri**	J	-	0.108*	0.670*	0.222*
†*Scapanorhynchus lewisii*	C	Cappetta, 1980, pl. 9, fig. 1	0.175	0.507	0.318
†*Aquilolamna milarcae*	C	Vullo et al., 2021, fig. 1B	0.067	0.624	0.309
†*Ptychodus* sp. 1	C	Vullo et al., 2024, fig. 1a, b; table S1	0.127	0.686	0.187
†*Ptychodus* sp. 2	C	Vullo et al., 2024, fig. 1c, d; table S1	0.124	0.708	0.168
†*Ptychodus* sp.*	C	-	0.126*	0.697*	0.177*
†*Bavariscyllium tischlingeri*	J	Thies and Leidner, 2011, pl. 44, fig. A	0.096	0.580	0.324
†*Palaeoscyllium formosum* 1	J	Kriwet and Klug, 2004, fig. 15a	0.123	0.658	0.219
†*Palaeoscyllium formosum* 2	J	Kriwet and Klug, 2004, fig. 15b	0.121	0.625	0.254
†*Palaeoscyllium formosum* 3	J	Kriwet and Klug, 2004, fig. 20a	0.132	0.644	0.224
†*Palaeoscyllium formosum**	J	-	0.125*	0.643*	0.232*

†*Palaeoscyllium minus*	J	Thies and Leidner, 2011, pl. 35, fig. A	0.121	0.626	0.253
†*Palaeoscyllium?* sp.	J	Thies and Leidner, 2011, pl. 52, fig. A	0.148	0.610	0.242
†*Paratriakis curtirostris*	C	Cappetta, 1980, pl. 24, fig. 1	0.130	0.711	0.159

APPENDIX 3.

List of all extant and extinct shark species examined in this study along with their higher taxonomy, maximum total length for extant taxa (mTL; in cm; based on Ebert et al., 2021, except *Dichichthys bigus*, which is based on White et al., 2024), neurocranial proportion (NP), trunk proportion (TP), caudal fin proportion (CP), and 'adjusted neurocranial proportion' (aNP) and 'adjusted caudal fin proportion' (aCP) (parts of data based on Appendices 1 and 2; see Figure 1C and text). Order codes: CAR: Carcharhiniformes; ECH, Echinorhiniformes; HET, Heterodontiformes; HEX, Hexanchiformes; LAM, Lamniformes; ORE, Orectolobiformes; PRI, Pristiophoriformes; 'SQ', Squalomorphii (Order incertae sedis); SQL, Squaliformes; SQT, Squatiniformes; SYN, †Synechodontiformes.

Species name	Order: Family	mTL	NP	TP	CP	aNP	aCP
EXTANT							
Chlamydoselachus anguineus	HEX: Chlamydoselachidae	196	0.040	0.690	0.270	0.058	0.391
Heptranchias perlo	HEX: Hexanchidae	139	0.100	0.638	0.262	0.157	0.411
Hexanchus griseus	HEX: Hexanchidae	550	0.111	0.586	0.303	0.189	0.517
Notorynchus cepedianus	HEX: Hexanchidae	296	0.130	0.558	0.312	0.233	0.559
Echinorhinus cookei	ECH: Echinorinidae	450	0.136	0.635	0.229	0.214	0.361
Cirrhigaleus barbifer	SQL: Squalidae	126	0.121	0.720	0.159	0.168	0.221
Squalus mitsukurii	SQL: Squalidae	125	0.155	0.643	0.202	0.241	0.314
Centrophorus granulosus	SQL: Centrophoridae	176	0.168	0.659	0.173	0.255	0.263
Deania calcea	SQL: Centrophoridae	162	0.188	0.599	0.213	0.314	0.356
Aculeola nigra	SQL: Etmopteridae	67	0.070	0.725	0.205	0.097	0.283
Centroscyllium excelsum	SQL: Etmopteridae	64	0.149	0.570	0.281	0.261	0.493
Etmopterus lucifer	SQL: Etmopteridae	47	0.151	0.614	0.235	0.246	0.383
Trigonognathus kabeyai	SQL: Etmopteridae	54	0.104	0.732	0.164	0.104	0.224
Centroscymnus owstonii	SQL: Somniosidae	120	0.118	0.618	0.194	0.191	0.314
Centroselachus crepidater	SQL: Somniosidae	105	0.061	0.709	0.230	0.086	0.324
Scymnodalatias sherwoodi	SQL: Somniosidae	85	0.061	0.703	0.236	0.087	0.336
Somniosus microcephalus	SQL: Somniosidae	756	0.085	0.711	0.204	0.120	0.287
Zameus squamulosus	SQL: Somniosidae	84	0.138	0.680	0.182	0.203	0.268
Oxynotus bruniensis	SQL: Oxynotidae	91	0.124	0.662	0.214	0.187	0.323
Dalatias licha	SQL: Dalatiidae	182	0.126	0.659	0.215	0.191	0.326
Euprotomicrus bispinatus	SQL: Dalatiidae	27	0.081	0.772	0.147	0.105	0.190
Heteroscymnoides marleyi	SQL: Dalatiidae	37	0.200	0.596	0.204	0.336	0.342
Isistius brasiliensis	SQL: Dalatiidae	56	0.115	0.729	0.156	0.158	0.214
Mollisquama sp.*	SQL: Dalatiidae	40	0.071	0.688	0.241	0.103	0.350
Squaliolus laticaudus	SQL: Dalatiidae	28	0.196	0.656	0.148	0.299	0.226
Pliotrema annae	PRI: Pristiophoridae	98	0.255	0.587	0.158	0.434	0.269
Pliotrema kajae	PRI: Pristiophoridae	143	0.304	0.550	0.146	0.553	0.265
Pliotrema warreni	PRI: Pristiophoridae	136	0.329	0.501	0.170	0.657	0.339
Pristiophorus japonicus	PRI: Pristiophoridae	153	0.288	0.540	0.172	0.533	0.319
Pristiophorus nudipinnis	PRI: Pristiophoridae	124	0.236	0.588	0.176	0.401	0.299
Squatina africana	SQT: Squatinidae	122	0.095	0.778	0.127	0.122	0.163

Species name	Order: Family	mTL	NP	TP	CP	aNP	aCP
Squatina japonica	SQT: Squatinidae	200	0.100	0.765	0.135	0.131	0.176
Heterodontus francisci	HET: Heterodontidae	122	0.119	0.661	0.220	0.180	0.333
Heterodontus zebra	HET: Heterodontidae	122	0.143	0.679	0.178	0.211	0.262
Cirrhoscyllium japonicum	ORE: Parascylliidae	49	0.086	0.689	0.225	0.125	0.327
Parascyllium ferrugineum	ORE: Parascylliidae	82	0.081	0.743	0.176	0.109	0.237
Brachaelurus waddi	ORE: Brachaeluridae	120	0.114	0.656	0.230	0.174	0.351
Eucrossorhinus dasypogon	ORE: Orectolobidae	125	0.187	0.614	0.199	0.305	0.324
Orectolobus ornatus	ORE: Orectolobidae	120	0.115	0.674	0.211	0.171	0.313
Orectolobus wardi	ORE: Orectolobidae	63	0.115	0.694	0.191	0.166	0.275
Sutorectus tentaculatus	ORE: Orectolobidae	92	0.129	0.650	0.221	0.198	0.340
Chiloscyllium plagiosum	ORE: Hemiscylliidae	95	0.108	0.695	0.197	0.155	0.283
Hemiscyllium freycineti	ORE: Hemiscylliidae	69	0.095	0.729	0.176	0.130	0.241
Stegostoma tigrinum	ORE: Stegostomatidae	354	0.117	0.414	0.468	0.283	1.130
Pseudoginglymostoma brevicaudatum	ORE: Ginglymostomatidae	75	0.092	0.655	0.253	0.140	0.386
Ginglymostoma cirratum	ORE: Ginglymostomatidae	308	0.123	0.593	0.284	0.207	0.479
Rhincodon typus	ORE: Rhincodontidae	2100	0.080	0.692	0.228	0.116	0.329
Mitsukurina owstoni	LAM: Mitsukurinidae	620	0.205	0.478	0.317	0.429	0.663
Carcharias taurus	LAM: Carchariidae	325	0.105	0.631	0.264	0.166	0.418
Odontaspis ferox	LAM: Odontaspididae	450	0.131	0.607	0.262	0.216	0.432
Odontaspis noronhai	LAM: Odontaspididae	427	0.122	0.606	0.272	0.201	0.449
Pseudocarcharias kamoharai	LAM: Pseudocarchariidae	122	0.126	0.662	0.212	0.190	0.320
Megachasma pelagios	LAM: Megachasmidae	820	0.097	0.588	0.315	0.165	0.536
Cetorhinus maximus	LAM: Cetorhinidae	1097	0.092	0.714	0.194	0.129	0.272
Alopias pelagicus	LAM: Alopiidae	428	0.059	0.400	0.541	0.148	1.353
Alopias superciliosus	LAM: Alopiidae	484	0.071	0.501	0.428	0.142	0.854
Alopias vulpinus	LAM: Alopiidae	575	0.056	0.416	0.525	0.135	1.262
Carcharodon carcharias	LAM: Lamnidae	640	0.115	0.694	0.191	0.166	0.275
Isurus oxyrinchus	LAM: Lamnidae	445	0.129	0.695	0.176	0.186	0.253
Isurus paucus	LAM: Lamnidae	430	0.108	0.681	0.211	0.159	0.310
Lamna ditropis	LAM: Lamnidae	305	0.107	0.695	0.198	0.154	0.285
Lamna nasus	LAM: Lamnidae	365	0.111	0.668	0.221	0.166	0.331
Apristurus laurussonii	CAR: Pentanchidae	76	0.210	0.530	0.260	0.396	0.491
Asymbolus vincenti	CAR: Pentanchidae	61	0.100	0.662	0.238	0.151	0.360
Bythaelurus canescens	CAR: Pentanchidae	73	0.127	0.640	0.233	0.198	0.364
Cephalurus cephalus/'sp.'	CAR: Pentanchidae	37	0.120	0.617	0.263	0.194	0.426
Figaro boardmani	CAR: Pentanchidae	61	0.128	0.656	0.216	0.195	0.329
Galeus arae	CAR: Pentanchidae	33	0.134	0.591	0.275	0.227	0.465
Halaelurus buergeri	CAR: Pentanchidae	49	0.114	0.673	0.213	0.169	0.316
Haploblepharus fuscus	CAR: Pentanchidae	69	0.097	0.698	0.205	0.139	0.294
Holohalaelurus regani	CAR: Pentanchidae	69	0.102	0.672	0.226	0.152	0.336
Parmaturus pilosus	CAR: Pentanchidae	64	0.100	0.678	0.222	0.147	0.327
Parmaturus xaniurus	CAR: Pentanchidae	61	0.126	0.652	0.222	0.193	0.340
Pentanchus profundicolus	CAR: Pentanchidae	51	0.141	0.532	0.327	0.265	0.615
Dichichthys bigus	CAR: Dichichthyidae	105	0.137	0.717	0.146	0.191	0.204

Species name	Order: Family	mTL	NP	TP	CP	aNP	aCP
Atelomycterus marmoratus	CAR: Scyliorhinidae	70	0.098	0.697	0.205	0.141	0.294
Aulohalaelurus labiosus	CAR: Scyliorhinidae	67	0.097	0.679	0.224	0.143	0.330
Cephaloscyllium ventriosum	CAR: Scyliorhinidae	100	0.120	0.666	0.214	0.180	0.321
Poroderma africanum	CAR: Scyliorhinidae	109	0.106	0.680	0.214	0.156	0.315
Schroederichthys chilensis	CAR: Scyliorhinidae	70	0.112	0.700	0.188	0.160	0.269
Schroederichthys maculatus	CAR: Scyliorhinidae	35	0.106	0.704	0.190	0.151	0.270
Scyliorhinus retifer	CAR: Scyliorhinidae	59	0.143	0.646	0.211	0.221	0.327
Ctenacis fehlmanni	CAR: Proscylliidae	46	0.124	0.648	0.228	0.191	0.352
Eridacnis barbouri	CAR: Proscylliidae	34	0.136	0.573	0.291	0.237	0.508
Proscyllium habereri	CAR: Proscylliidae	65	0.102	0.679	0.219	0.150	0.323
Gollum attenuatus	CAR: Pseudotriakidae	110	0.159	0.653	0.188	0.243	0.288
Planonasus parini	CAR: Pseudotriakidae	53	0.098	0.688	0.214	0.142	0.311
Pseudotriakis microdon	CAR: Pseudotriakidae	296	0.109	0.689	0.202	0.158	0.293
Leptocharias smithii	CAR: Leptochariidae	82	0.115	0.649	0.236	0.177	0.364
Furgaleus macki	CAR: Triakidae	160	0.153	0.658	0.189	0.233	0.287
Gogolia filewoodi	CAR: Triakidae	74	0.186	0.591	0.223	0.315	0.377
Galeorhinus galeus	CAR: Triakidae	195	0.170	0.599	0.231	0.284	0.386
Hemitriakis japanica	CAR: Triakidae	120	0.126	0.694	0.180	0.182	0.259
Hypogaleus hypugaensis	CAR: Triakidae	150	0.098	0.675	0.227	0.145	0.336
Iago omanesis	CAR: Triakidae	89	0.116	0.654	0.230	0.177	0.352
Mustelus californicus	CAR: Triakidae	125	0.091	0.709	0.200	0.128	0.282
Mustelus fasciatus	CAR: Triakidae	177	0.150	0.624	0.226	0.240	0.362
Mustelus higmani	CAR: Triakidae	88	0.129	0.681	0.190	0.189	0.279
Mustelus lenticulatus	CAR: Triakidae	151	0.126	0.669	0.205	0.188	0.306
Mustelus lunulatus	CAR: Triakidae	175	0.115	0.668	0.217	0.172	0.325
Mustelus mosis	CAR: Triakidae	150	0.122	0.689	0.189	0.177	0.274
Mustelus whitneyi	CAR: Triakidae	87	0.123	0.683	0.194	0.180	0.284
Scylliogaleus quecketti	CAR: Triakidae	102	0.106	0.743	0.151	0.143	0.203
Triakis acutipinna	CAR: Triakidae	102	0.102	0.688	0.210	0.148	0.305
Triakis maculata	CAR: Triakidae	240	0.137	0.651	0.212	0.210	0.326
Triakis megalopterus	CAR: Triakidae	208	0.111	0.673	0.216	0.165	0.321
Triakis scyllium	CAR: Triakidae	150	0.092	0.716	0.192	0.128	0.268
Triakis semifasciata	CAR: Triakidae	210	0.099	0.669	0.232	0.148	0.347
Chaenogaleus macrostoma	CAR: Hemigaleidae	125	0.105	0.674	0.221	0.156	0.328
Hemigaleus microstoma	CAR: Hemigaleidae	114	0.133	0.673	0.194	0.198	0.288
Hemipristis elongata	CAR: Hemigaleidae	240	0.094	0.671	0.235	0.140	0.350
Paragaleus tengi	CAR: Hemigaleidae	93	0.109	0.672	0.219	0.162	0.326
Carcharhinus amblyrhynchoides	CAR: Carcharhinidae	182	0.121	0.630	0.249	0.192	0.395
Carcharhinus amboinensis	CAR: Carcharhinidae	280	0.143	0.651	0.206	0.220	0.316
Carcharhinus borneensis	CAR: Carcharhinidae	65	0.153	0.571	0.276	0.268	0.483
Carcharhinus falciformis	CAR: Carcharhinidae	350	0.140	0.617	0.243	0.227	0.394
Carcharhinus galapagensis	CAR: Carcharhinidae	300	0.117	0.646	0.237	0.181	0.367
Carcharhinus hemiodon	CAR: Carcharhinidae	102	0.153	0.595	0.252	0.257	0.424
Carcharhinus isodon	CAR: Carcharhinidae	200	0.150	0.624	0.226	0.240	0.362
Carcharhinus limbatus	CAR: Carcharhinidae	286	0.120	0.653	0.227	0.184	0.348

Species name	Order: Family	mTL	NP	TP	CP	aNP	aCP
Carcharhinus longimanus	CAR: Carcharhinidae	395	0.127	0.579	0.294	0.219	0.508
Carcharhinus macloti	CAR: Carcharhinidae	110	0.163	0.597	0.240	0.273	0.402
Carcharhinus melanopterus	CAR: Carcharhinidae	180	0.145	0.624	0.231	0.232	0.370
Carcharhinus obscurus	CAR: Carcharhinidae	420	0.106	0.654	0.240	0.162	0.367
Carcharhinus porosus	CAR: Carcharhinidae	134	0.140	0.633	0.227	0.221	0.359
Carcharhinus sealei	CAR: Carcharhinidae	86	0.119	0.650	0.231	0.183	0.355
Carcharhinus sorrah	CAR: Carcharhinidae	166	0.120	0.628	0.252	0.182	0.401
Glyphis sp.**	CAR: Carcharhinidae	260	0.133	0.622	0.245	0.214	0.394
Isogomphodon oxyrhynchus	CAR: Carcharhinidae	244	0.185	0.564	0.251	0.328	0.445
Lamiopsis temmincki	CAR: Carcharhinidae	178	0.140	0.642	0.218	0.218	0.340
Loxodon macrorhinus	CAR: Carcharhinidae	99	0.102	0.633	0.265	0.161	0.419
Negaprion brevirostris	CAR: Carcharhinidae	368	0.111	0.669	0.220	0.166	0.329
Nasolamia velox	CAR: Carcharhinidae	165	0.179	0.565	0.256	0.317	0.453
Prionace glauca	CAR: Carcharhinidae	384	0.129	0.625	0.246	0.206	0.394
Rhizoprionodon acutus	CAR: Carcharhinidae	178	0.131	0.597	0.272	0.219	0.456
Rhizoprionodon lalandii	CAR: Carcharhinidae	102	0.147	0.597	0.256	0.246	0.429
Rhizoprionodon longurio	CAR: Carcharhinidae	154	0.174	0.595	0.231	0.292	0.388
Rhizoprionodon oligolinx	CAR: Carcharhinidae	88	0.130	0.624	0.246	0.208	0.394
Rhizoprionodon terraenovae	CAR: Carcharhinidae	113	0.125	0.635	0.240	0.197	0.378
Scoliodon laticaudus	CAR: Carcharhinidae	74	0.159	0.619	0.222	0.257	0.359
Triaenodon obesus	CAR: Carcharhinidae	213	0.098	0.651	0.251	0.151	0.386
Galeocerdo cuvier	CAR: Galeocerdidae	550	0.101	0.612	0.287	0.165	0.489
Eusphyra blochii	CAR: Sphyrnidae	186	0.072	0.601	0.327	0.120	0.544
Sphyrna lewini	CAR: Sphyrnidae	430	0.067	0.629	0.304	0.107	0.483
Sphyrna tiburo	CAR: Sphyrnidae	150	0.113	0.584	0.303	0.193	0.519
EXTINCT							
†*Paraorthacodus* sp.	SYN: †Paraorthacodontidae	-	0.135	0.503	0.362	0.268	0.720
†*Centrosqualus primaevus*	SQL: Squalidae	-	0.118	0.684	0.198	0.173	0.290
†*Cretascymnus adonis*	SQL: Somniosidae	-	0.140	0.723	0.137	0.194	0.190
†*Synechodus* sp.	SQT: †Palaeospinacidae	-	0.126	0.604	0.270	0.209	0.447
†*Pseudorhina acanthoderma*	SQT: Squatinidae	-	0.096	0.791	0.113	0.121	0.143
†*Pseudorhina alifera*	SQT: Squatinidae	-	0.091	0.735	0.174	0.124	0.237
†*Protospinax annectans*	'SQ': †Protospinacidae	-	0.160	0.685	0.155	0.234	0.226
†*Heterodontus zitteli*	HET: Heterodontidae	-	0.124	0.671	0.205	0.185	0.306
†*Paracestracion falcifer*	HET: †Paracestracionidae	-	0.129	0.679	0.192	0.190	0.283
†*Mesiteia emiliae*	ORE: †Mesiteiidae	-	0.082	0.774	0.144	0.106	0.186
†*Phorcynis catulina*	ORE: Family incertae sedis	-	0.118	0.664	0.218	0.178	0.328
†*Palaeocarcharias stromeri*	LAM: †Palaeocarchariidae***	-	0.108	0.670	0.222	0.161	0.331
†*Scapanorhynchus lewisii*	LAM: Mitsukurinidae	-	0.175	0.507	0.318	0.345	0.627
†*Aquilolamna milarcae*	LAM: †Aquilolamnidae	-	0.067	0.624	0.309	0.107	0.495
†*Ptychodus* sp.	LAM: †Ptychodontidae	-	0.126	0.697	0.177	0.181	0.254
†*Otodus megalodon*****	LAM: †Otodontidae	-	0.111	0.670	0.218	0.166	0.326
†*Bavariscyllium tischlingeri*	CAR: Scyliorhinidae	-	0.096	0.580	0.324	0.166	0.559
†*Palaeoscyllium formosum*	CAR: Scyliorhinidae	-	0.125	0.643	0.232	0.194	0.361

Species name	Order: Family	mTL	NP	TP	CP	aNP	aCP
†*Palaeoscyllium minus*	CAR: Scyliorhinidae	-	0.121	0.626	0.253	0.193	0.404
†*Palaeoscyllium*? sp.	CAR: Scyliorhinidae	-	0.148	0.610	0.242	0.243	0.397
†*Paratriakis curtirostris*	CAR: Triakidae	-	0.130	0.711	0.159	0.183	0.224

*Average PC value between *Mollisquama mississippiensis* (0.230) and *M. parini* (0.251).

**Average CP value among *Glyphis gangeticus* (0.242), *G. garricki* (0.238), and *G. glyphis* (0.255).

*** This familial assignment is questionable (see Villalobos-Segura et al., 2023).

**** Inferred values used for the cluster analysis (see text for explanation).
